# PHGDH loss promotes hypoxia tolerance through glycolytic reprogramming and enhanced HIF-1 activity

**DOI:** 10.1038/s41420-026-03194-9

**Published:** 2026-06-10

**Authors:** Eduard Petrosyan, Louise A. W. Martin, Isabelle Legge, John Walsby-Tickle, Adam C. Sedgwick, Mark A. Hill, James S. O. McCullagh, Monica M. Olcina, Ester M. Hammond

**Affiliations:** 1 Department of Oncology, The University of Oxford, Oxford, UK; 2 Chemistry Research Laboratory, Department of Chemistry, University of Oxford, Oxford, UK; 3 Department of Chemistry, King’s College London, London, UK

**Keywords:** Molecular biology, Cancer metabolism, Cancer microenvironment

## Abstract

Radiotherapy is widely used in the treatment of lung cancer; a number of intrinsic and extrinsic mechanisms of resistance exist, including hypoxia. Targeting metabolic pathways that support redox homeostasis has been proposed as a strategy to enhance radiosensitivity. The serine synthesis pathway enzyme, phosphoglycerate dehydrogenase (PHGDH), has been implicated in resistance to several anticancer therapies; however, its role in radiotherapy response is poorly defined. We show that PHGDH expression positively correlates with established hypoxia gene expression signatures in lung adenocarcinoma and squamous cell carcinoma patient datasets and is induced under hypoxia (<0.1% O_2_) in lung cancer cell lines. Hypoxic induction of PHGDH appears mediated by both HIF-1 and PERK signalling, linking PHGDH regulation to hypoxia and the unfolded protein response. Using CRISPR-Cas9 PHGDH knockout models, we demonstrate that loss of PHGDH does not enhance radiosensitivity in normoxia or hypoxia when extracellular serine and glycine are available at physiological or supraphysiological concentrations. Radiosensitisation is observed only under complete serine/glycine deprivation. Unexpectedly, PHGDH loss confers increased tolerance to hypoxic stress, associated with elevated glycolytic flux, increased lactate production, accelerated HIF-1α stabilisation and enhanced expression of hypoxia-inducible genes. Overall, these data indicate that targeting PHGDH alone is unlikely to impact radioresistance in lung cancer under physiologically relevant nutrient conditions and may instead promote metabolic adaptation to hypoxia. This highlights the importance of microenvironmental context when evaluating metabolic targets for combination with radiotherapy.

## Introduction

Lung cancer is the leading cause of cancer-related deaths globally, accounting for 1.8 million deaths in 2022 [1]. A significant proportion of lung cancer patients receive radiotherapy with curative intent, although this is determined by factors including disease stage, patient fitness, comorbidities and access to facilities [2]. Hypoxia, or lack of oxygen, is a common hallmark of lung cancers, arising from an imbalance between elevated proliferation rates and insufficient vasculature. Importantly, hypoxia is a major cause of radioresistance, as oxygen is required to stabilise radiation-induced DNA damage [3]. Consequently, significant effort has been spent identifying agents that improve the efficacy of radiotherapy (radiosensitisers), with a particular focus on those agents that are effective in hypoxic conditions [4].

Serine and glycine synthesis pathways play an important role in the metabolic reprogramming of cancer cells and response to therapy. Serine and glycine are crucial for upregulation of cellular pathways that drive cell proliferation, including nucleotide synthesis through one-carbon metabolism. Serine is synthesised de novo through the serine synthesis pathway (SSP), which includes 3-phosphoglycerate dehydrogenase (PHGDH) as a rate-limiting step. PHGDH catalyses the conversion of 3-phosphoglycerate (3-PG), a glycolytic intermediate, into 3-phosphohydroxypyruvate (3-PHP). The subsequent two steps of the SSP are catalysed by enzymes phosphoserine aminotransferase 1 (PSAT1) and phosphoserine phosphatase (PSPH) [5, 6]. Serine can then be converted into glycine through a reaction catalysed by serine hydroxymethyltransferase (SHMT1/2) enzymes [7]. Importantly, serine contributes to glutathione (GSH) production by serving as a precursor for glycine via these SHMT1/2-mediated reactions [8]. GSH is critical to antioxidant defense, and without sufficient levels, an increase in reactive oxygen species (ROS) occurs [9, 10].

Evidence across multiple cancer types indicates that PHGDH promotes resistance to chemotherapy and targeted agents, whereas genetic or pharmacological inhibition enhances treatment sensitivity. For example, PHGDH upregulation has been demonstrated to contribute to acquired resistance to erlotinib in EGFR-mutant lung adenocarcinoma cells [11]. Repression of PHGDH has been shown to sensitise triple-negative breast cancer cells to doxorubicin by increasing oxidative stress and apoptosis [12]. Most recently, high PHGDH expression was shown to promote 5-fluorouracil chemoresistance in colorectal cancer models by upregulating pro-survival Hedgehog signalling [13].

The role of PHGDH in radiotherapy response is less well-characterised. A recent study demonstrated that both knockdown and pharmacological inhibition of PHGDH increased the radiosensitivity of glioblastoma stem cells through ROS-mediated DNA damage [14]. Furthermore, inhibition of PHGDH has been shown to radiosensitise colorectal cancer cells in hypoxic ( < 0.1% O_2_) conditions, again through disrupting the redox balance and elevating ROS levels [15]. Inhibition of the serine/glycine pathway has been more widely investigated. Notably, targeting SHMT with sertraline resulted in increased sensitivity to radiation in non-small cell lung cancer (NSCLC) and was attributed to decreased GSH and increased ROS [16]. Further support for targeting serine and glycine metabolism comes from evidence that dietary restriction of serine/glycine enhances radiosensitivity in breast, colorectal and murine pancreatic cancer models [17].

PHGDH expression has not been characterised in hypoxic conditions, however it was shown to be a transcriptional target of Activating Transcription Factor 4 (ATF4) in response to serum starvation [18]. ATF4 plays a pivotal role in the unfolded protein response (UPR), which, in addition to being induced in response to starvation, is also active in the hypoxic conditions most associated with radiation resistance [19, 20]. GSH levels increase in response to hypoxia, which also suggests a possible dependence on serine/glycine levels [10]. Together, these findings led us to hypothesise that targeting PHGDH could increase radiosensitivity and, most importantly, that this could combat hypoxia-mediated radiation resistance in lung cancer.

## Results

### PHGDH is hypoxia-inducible

**Fig. 1:**
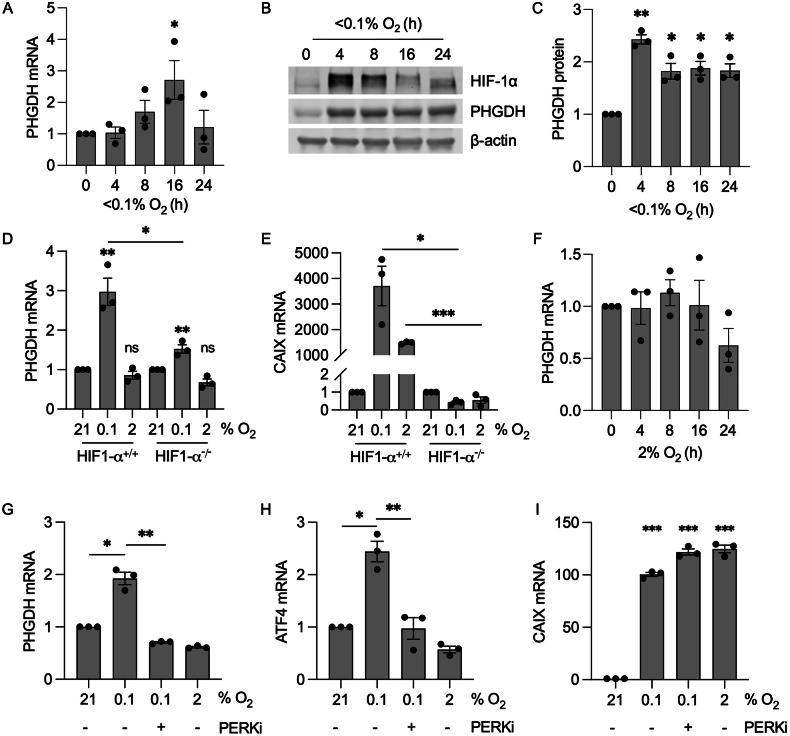
Hypoxia-mediated induction of PHGDH is dependent on HIF-1 and the UPR. **A** A549 cells were treated with <0.1% O_2_ for 0–24 h and RT-qPCR for PHGDH was carried out. The levels of CAIX in the same samples are shown in Fig. S2A. **B** A549 cells were exposed to <0.1% O_2_ for the times indicated, followed by western blotting for PHGDH, HIF-1α and β-actin as a loading control. **C** Quantification of changes in PHGDH from western blots generated in part B. Band intensities were normalised to β-actin loading control. **D, E** HIF-1α wild-type and knock-out RKO cells were exposed to 21, 2, or <0.1% O_2_ for 16 h and PHGDH (**D**) and CAIX (**E**) expression levels were determined using RT-qPCR. **F**. A549 cells were treated with <2% O_2_ for 0–24 h and RT-qPCR for PHGDH was carried out. **G–I** A549 cells were exposed to 21, 2 or <0.1% O_2_ for 16 h with or without AMG PERK 44 (20 μM). RT-qPCR for PHGDH (**G**), ATF4 (**H**) and CAIX (**I**) was carried out. Data represent the mean of three replicates and error bars indicate the SEM. For statistical analysis, a two-tailed Student *t test* was performed. *p < *0.05*, **p < 0.01, ***p < *0.001*, ns nonsignificant.

To investigate the potential relationship between expression of the SSP and hypoxia in lung cancer, we began by studying the expression of PHGDH, PSAT1 and PSPH in normal tissue versus tumour. In lung squamous cell carcinoma (LUSC), all three SSP genes showed significantly higher expression in tumour vs normal, whilst in lung adenocarcinoma (LUAD), only PSAT1 was significantly different (Fig. S1A, B). Next, we looked for correlation between PHGDH/PSAT1/PSPH mRNA levels and established hypoxia gene expression signatures in the TCGA lung patient datasets [21, 22]. PHGDH, PSAT1 and PSPH showed a positive correlation with two hypoxia signatures in LUSC and LUAD, suggesting that the SSP could be hypoxia-inducible in patients with lung cancer (Fig. S1C–F) [23, 24]. Given the druggable nature of PHGDH, we sought to confirm the relationship between expression and hypoxia in vitro. A549 cells were exposed to hypoxia and RT-qPCR was carried out using the well-characterised HIF target gene, carbonic anhydrase IX (CAIX) as a positive control [25]. As expected, both PHGDH and CAIX levels were significantly increased in hypoxia (Figs. 1A and S2A). Hypoxia-mediated induction of PHGDH was also determined in a second cell line, H460 (Fig. S2B, C). PHGDH protein levels were found to increase earlier than the observed change in mRNA, reaching maximum levels after just 4 h in hypoxia ( < 0.1% O_2_) (Fig. 1B, C). Notably, no significant change in PHGDH protein was determined in H460 cells exposed to hypoxia, although we did note that they appeared to have higher baseline expression compared to A549 cells (Fig. S2D, E).

The positive correlation between PHGDH expression and the hypoxia signatures suggested to us that PHGDH could be a target of the HIF transcription factors. To determine if PHGDH was regulated by HIF-1, PHGDH mRNA was measured in genetically matched cell lines RKO and RKO^HIF-1α-/-^ under normoxia and hypoxia ( < 0.1% O_2_ and 2% O_2_). PHGDH induction was again observed in hypoxia ( < 0.1% O_2_) and was significantly reduced, but not entirely abrogated, in the absence of HIF-1α. Surprisingly, PHGDH was not induced in response to 2% O_2_, where a robust induction of CAIX was observed (Fig. 1D, E). To exclude the possibility that PHGDH was induced with different kinetics in 2% O_2,_ further analysis was carried out. However, no significant change in PHGDH expression was determined over the time period examined (0–24 h) (Fig. 1F). Together, these data suggested that the induction of PHGDH in hypoxia is not entirely HIF-1 mediated and is dependent on the exact level of oxygen. PHGDH expression has been linked to the UPR, specifically via PERK-ATF4 signalling [26, 27]. Given the established links between hypoxia and the UPR [28], we asked whether inhibiting PERK would influence PHGDH expression under hypoxic conditions. A549 cells were treated with PERKi (AMG PERK 44) and exposed to hypoxia, followed by RT-qPCR for PHGDH, CAIX and ATF4 (Fig. 1G–I). Again, PHGDH was not induced in response to 2% O_2_ but did increase in <0.1% O_2_. Somewhat surprisingly, inhibition of PERK completely abrogated the induction of PHGDH and ATF4 in <0.1% O_2,_ whilst it did not affect CAIX expression. Together, these data suggest that PHGDH is regulated by both HIF-1 and the PERK-mediated UPR in hypoxic conditions, or that in this context PERK signalling controls HIF-1 activity. Interestingly, potential binding sites for both HIF-1 and ATF4 have been identified in the PHGDH promoter sequence (Fig. S2F, G).

**Fig. 2:**
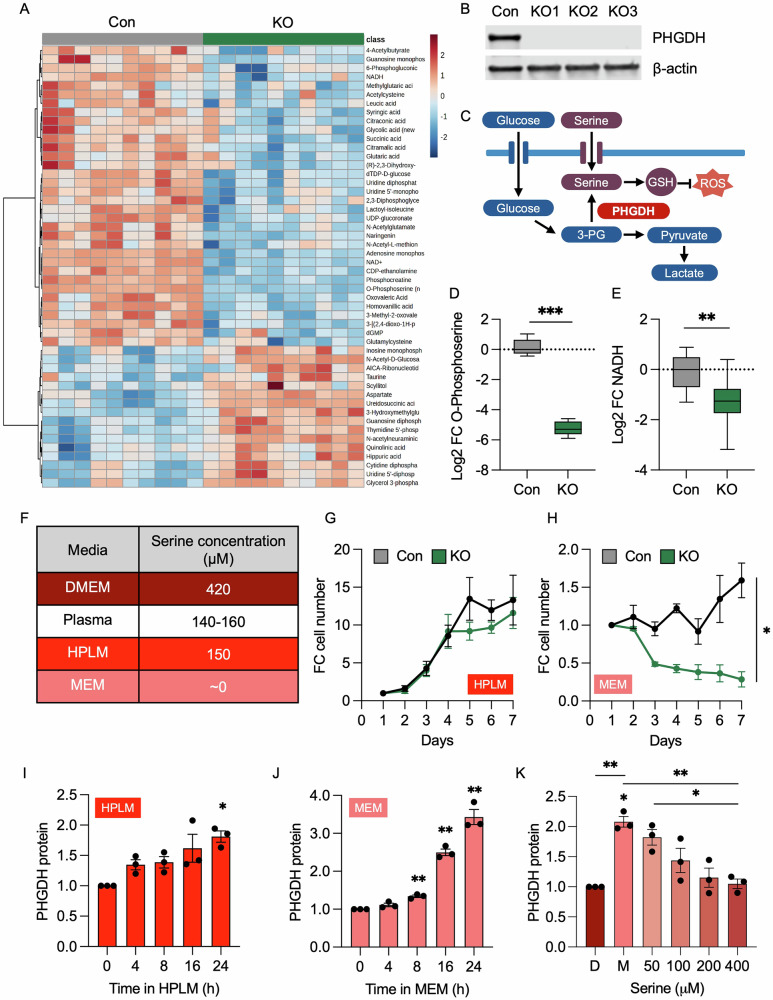
PHGDH-dependent control of metabolic state and proliferation. **A** IC-MS metabolomics heatmap showing z-scored relative metabolite abundances in parental control (Con) and PHGDH KO1 A549 cells grown in normoxia (21% O_2_), with metabolites hierarchically clustered and samples grouped by condition. **B** Validation of PHGDH knock-out in three A549 CRISPR-Cas9 clones (KO1, KO2, KO3). A549 cells were used as a parental control (Con). **C** Schematic representation of the de novo serine synthesis pathway (SSP). Comparisons of the levels of O-Phosphoserine (**D**), NADH (**E**) between A549 and PHGDH KO1 cells under normoxia (21% O_2_). **F** The levels of serine (μM) in each of the media used compared to normal plasma [57]. **G** Proliferation of parental and PHGDH KO1 cells was measured over 7 days of growth in HPLM. Fold change relative to the first day post-seeding was calculated. **H** Proliferation of parental and PHGDH KO1 cells was measured over 7 days of growth in MEM. Fold change relative to the first day post-seeding was calculated. **I** Quantification of PHGDH protein expression in parental A549 cells cultured in HPLM for the indicated times. Band intensity values were normalised to β-actin loading control and are relative to the 0 h condition. A representative western blot is shown in Fig. S3G. **J** Quantification of PHGDH protein expression in parental A549 cells cultured in MEM for the indicated times. Band intensity values were normalised to β-actin loading control and are relative to the 0 h condition. A representative western blot is shown in Fig. S3H. **K** Quantification of PHGDH protein levels in parental A549 cells cultured in DMEM (D), MEM (M), or MEM supplemented with increasing concentrations of serine (50–400 µM). Band intensity values were normalised to β-actin loading control and expressed relative to the DMEM condition. A representative western blot is shown in Fig. S3I. Data represent the mean of three replicates and error bars indicate the SEM. For statistical analysis of western blot data, a two-tailed unpaired Student *t* *test* was performed. For proliferation data, a two-tailed paired Student *t-test* was used. *p < *0.05*, **p < 0.01, ***p < *0.001*.

To test our hypothesis that targeting PHGDH could increase radiosensitivity, we generated CRISPR-Cas9 PHGDH knock-out (KO) cells. As expected, ion chromatography mass spectrometry (IC-MS) analysis of anionic metabolites in parental A549 and PHGDH KO cells revealed predicted changes in serine metabolism (Figs. 2A–C and S3A–C) [29, 30]. For example, a significant reduction in O-Phosphoserine levels was observed in PHGDH KO cells, confirming the abrogation of the SSP (Fig. 2D). Levels of NADH, the reduced form of the PHGDH co-factor NAD^+^, were also significantly lower in the KO cells (Fig. 2E). However, despite a complete loss of PHGDH and therefore SSP, there was no significant change in proliferation rate (Fig. S3D). However, it is important to note that although PHGDH is essential for de novo serine synthesis, serine can also be imported from the media [31]. Standard tissue culture media contain supraphysiological levels of serine (DMEM - 420 μM vs 140–160 μM in human plasma) [32–34]. This readily available excess serine means that cells grown in DMEM are not dependent on the SSP. Therefore, we performed experiments using a range of media containing different serine concentrations: DMEM (420 μM serine), HPLM (150 μM serine) and MEM ( ~ 0 μM serine) (Fig. 2F and Table S3 for full description of the media). No PHGDH-dependent effect on proliferation rate was determined when cells were grown in HPLM; however, in MEM, cells lacking PHGDH grew significantly slower (Figs. 2G, H and S3E, F). PHGDH expression was found to increase when A549 cells were grown in the media with either physiological levels of serine (HPLM) or no serine (MEM) (Figs. 2I, J and S3G, H). To further verify the relationship between the expression of the SSP and exogenous serine levels, MEM was supplemented with increasing concentrations of serine. As previously, PHGDH levels increased in MEM, but this effect was lost as serine levels increased and was completely abrogated when the levels of serine found in DMEM (400 μM) were reached (Figs. 2K and S3I).

### Loss of PHGDH does not increase radiosensitivity

**Fig. 3:**
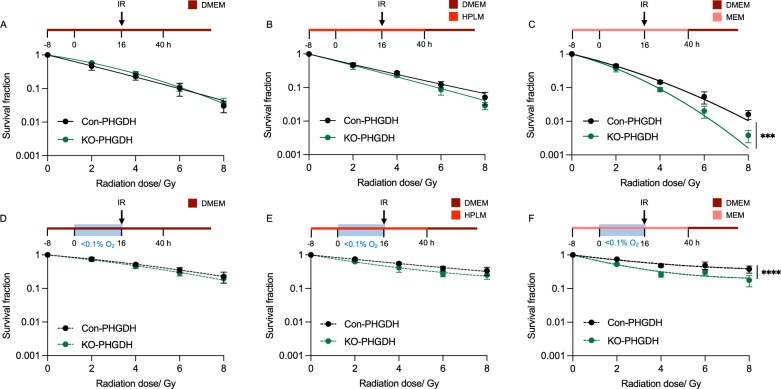
The effect of PHGDH KO on radiosensitivity in normoxia and hypoxia. A549 (parental and PHGDH KO1) cells were irradiated (0–8 Gy) in normoxia (21% O_2_) followed by a colony survival assay. Prior to and during irradiation, cells were either grown in DMEM (**A**), HPLM (**B**) or MEM (**C**). 24 h after irradiation, all media were replaced with fresh DMEM. A schematic of the experimental setup is indicated. A549 (parental and PHGDH KO1) cells were irradiated (0–8 Gy) in hypoxia ( < 0.1% O_2_) followed by a colony survival assay. Prior to and during irradiation cells were either grown in DMEM (**D**), HPLM (**E**) or MEM (**F**). 16 h before irradiation, cells were transferred to hypoxia ( < 0.1% O_2_) and were irradiated in hypoxic conditions. Cells were returned to normoxia shortly after irradiation. 24 h after irradiation, all media was replaced with fresh DMEM. A schematic of the experimental set-up is indicated. Data represent the mean of three replicates and error bars indicate the SEM. For statistical analysis, survival curves were fitted using a linear–quadratic model and compared by an extra-sum-of-squares F test. ***p < 0.001, ****p < *0.0001*.

**Fig. 4:**
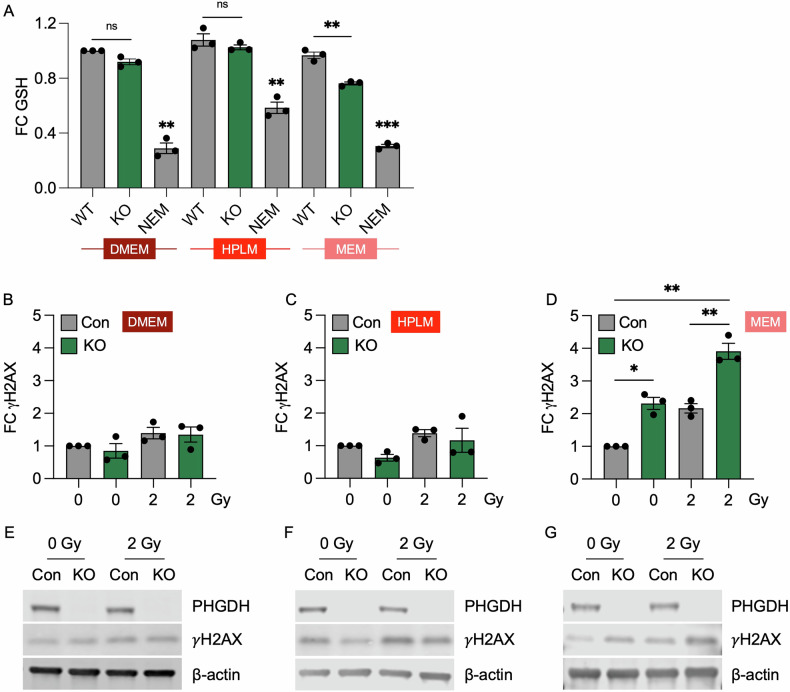
Loss of PHGDH in the absence of extracellular serine results in decreased GSH levels and increased DNA damage. **A** Parental control and PHGDH KO1 A549 cells were grown in DMEM, HPLM and MEM for 16 h, under normoxia. Fold change in GSH levels was calculated in relation to baseline levels in cells cultured in DMEM. A549 cells were also treated with N-ethylmaleimide (NEM) (200 μM, 6 h) in each media type to reduce GSH, as a negative control. A549 (parental and PHGDH KO1) cells were grown in the media indicated DMEM (**B**), HPLM (**C**) and MEM (**D**) for 24 h followed by irradiation (2 Gy). Western blotting and quantification of changes in γH2AX relative to β-actin was carried out. Fold change relative to non-irradiated control cells (0 Gy, Con) is shown. Representative western blots are shown in (**E–G**). Data represent the mean of three replicates and error bars indicate the SEM. For statistical analysis, a two-tailed Student’s *t-test* was performed. *p < *0.05*, **p < 0.01, ***p < *0.001*.

The cell lines (parental and KO1) were grown in the range of media (DMEM, HPLM and MEM) and irradiated (0–8 Gy) before allowing colonies to form. In agreement with a previous report, cells grown in MEM, i.e. the absence of serine/glycine, were significantly more sensitive to radiation than cells grown in DMEM or HPLM (Fig. S4A) [17]. Loss of PHGDH did not have a significant effect on radiosensitivity in DMEM or HPLM media (Fig. 3A, B). However, when irradiated in MEM, the PHGDH KO cells (KO1 and KO2) showed significantly increased radiosensitivity (Figs. 3C and S4B). Next, we asked how radiosensitivity in hypoxia was affected by the loss of PHGDH. As expected, cells irradiated in hypoxic conditions ( < 0.1% O_2_) were more radioresistant than those treated in normoxia with oxygen enhancement ratios (OER) of 2–3.5 (Fig. S4C–E). However, as determined in normoxia loss of PHGDH only led to enhanced radiosensitivity when grown in MEM in hypoxia (Fig. 3D–F). To investigate further, we determined changes in GSH levels in each media type and found that a PHGDH-dependent difference in GSH was only significant when the cells were grown in MEM (Fig. 4A). As expected, regardless of the media the cells were grown in, GSH levels increased in response to hypoxia indicating that even when grown in MEM, there are other cellular mechanisms to generate GSH. The hypoxia-mediated increase in GSH was not PHGDH dependent. As seen in normoxia, loss of PHGDH only reduced GSH in hypoxia, compared to the parental cells, when serine and glycine were also missing from the media, i.e. cells were grown in MEM (Fig. S5A). Next, we investigated the levels of DNA damage, determined using γH2AX and found that PHGDH KO1 cells had both higher levels of γH2AX at baseline and post radiation (2 Gy) when grown in MEM but not DMEM or HPLM (Fig. 4B–G and KO2 and KO3 Fig. S5B–D). Overall, our data shows that loss of PHGDH only leads to significant radiosensitisation when cells are completely deprived of extracellular serine/glycine. This radiosensitisation effect is potentially due to reduced anti-oxidant capacity and higher levels of baseline DNA damage in the absence of PHGDH.

### Loss of PHGDH leads to higher glycolytic rates and elevated levels of lactate

**Fig. 5:**
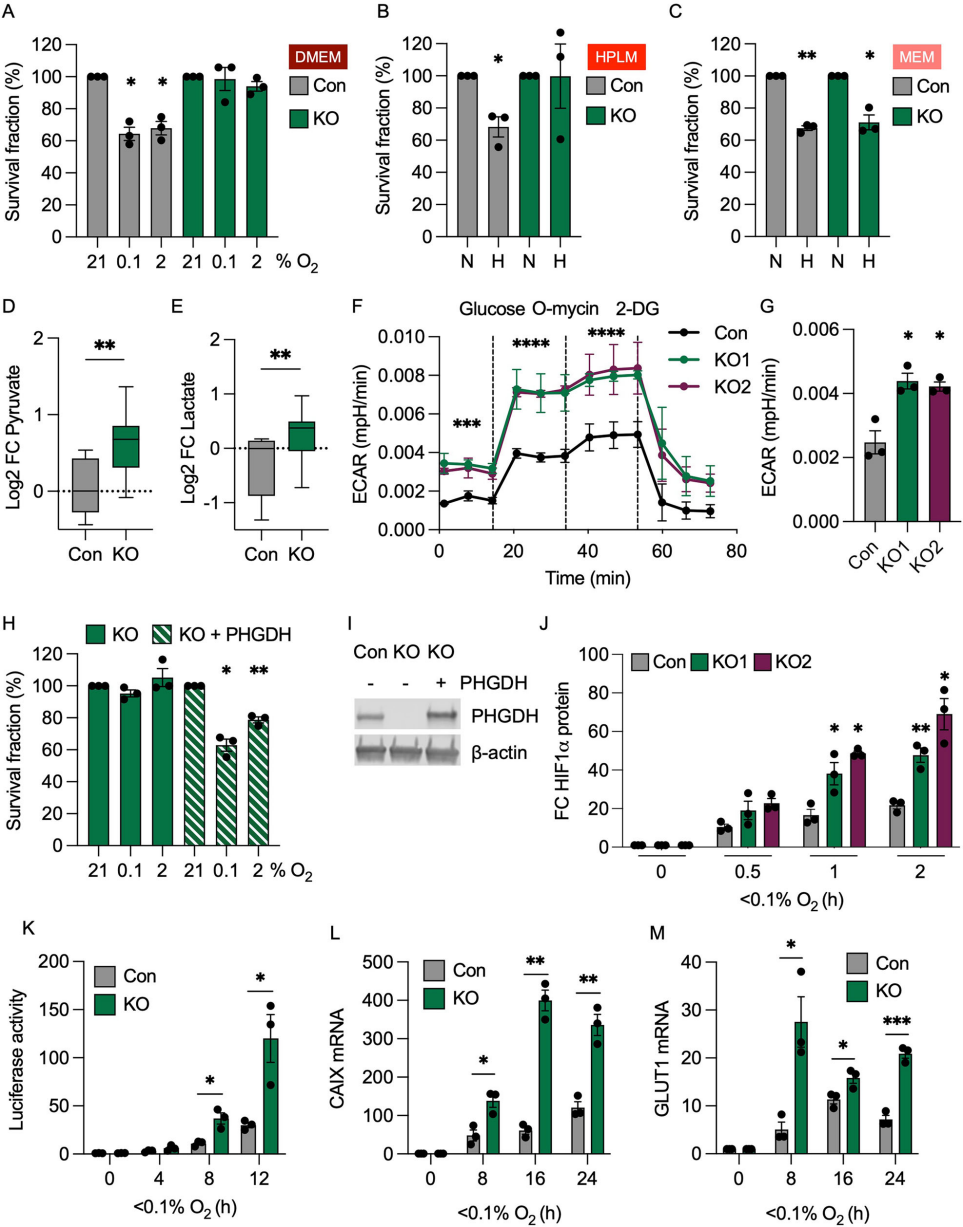
Loss of PHGDH leads to improved survival in hypoxic conditions. **A** A549 (Con and PHGDH KO1) grown in DMEM were exposed to hypoxia ( < 0.1 or 2% O_2_) for 16 h. Cells were returned to normoxic conditions and colonies allowed to form. Colony survival is shown as a per cent survival fraction relative to the 21% O_2_ control. **B** Parental and PHGDH KO1 A549 cells were grown in HPLM media for 45 h. After 5 h in HPLM, cells were exposed to hypoxia (0.1% O_2_) for 16 h. Following the hypoxia exposure, cells were kept in HPLM for an additional 24 h and then returned to DMEM and grown under normoxia to allow colony formation. **C** Parental and PHGDH KO1 A549 cells were grown in MEM media for 45 h. After 5 h in MEM, cells were exposed to hypoxia (0.1% O_2_) for 16 h. Following the hypoxia exposure, cells were kept in MEM for an additional 24 h and then returned to DMEM and grown under normoxia to allow colony formation. Comparisons of the levels of pyruvate (**D**), lactate (**E**) between A549 and PHGDH KO1 cells under normoxia (21% O_2_). **F** Glycolytic stress test on parental (Con) and PHGDH KO1 and KO2 A549 cells. **G** Using the data from part F, glycolysis levels were calculated based on the extracellular acidification rates (ECAR) in parental (Con) and KO1 and KO2 cell lines. **H** PHGDH KO1 cells expressing an empty vector control or PHGDH overexpression vector (KO1 + PHGDH) were cultured in DMEM, under normoxia and hypoxia ( < 0.1 or 2% O_2_) for 16 h. Cells were returned to normoxic conditions and colonies allowed to form. **I** Representative western blot showing the expression of PHGDH in parental control, PHGDH KO1 and PHGDH overexpressing cells. β-actin was used as a loading control. **J** Quantification of HIF-1α protein levels in parental control (Con) and PHGDH KO clones (KO1 and KO2) following exposure to hypoxia ( < 0.1% O_2_) for the indicated times. Band intensity values were normalised to β-actin and expressed as fold change relative to normoxic Con cells (0 h, Con). A representative western blot is shown in Fig. S7A. **K** A549 cells and PHGDH KO1 cells were transfected with an HRE-Luciferase reporter and then exposed to hypoxia ( < 0.1% O_2_) for 0–12 h. Relative luciferase levels are shown. A549 and PHGDH KO1 cells were exposed to hypoxia ( < 0.1% O_2_) for the times indicated, followed by qPCR for CAIX (**L**) and GLUT1 (**M**). Data represent the mean of three replicates and error bars show the standard error of the mean (SEM). An ordinary one-way ANOVA test was performed for statistical analysis of the extracellular flux analysis data. Elsewhere, a two-tailed Student *t-test* was performed. *p < *0.05*, **p < 0.01, ***p < *0.001*.

Whilst determining relative radiosensitivity, we noted that cells lacking PHGDH appeared to survive better in hypoxic conditions. To formally investigate, we exposed the PHGDH KO1 cells and parental control to hypoxia ( < 0.1 and 2% O_2_) and carried out a colony survival assay. While exposure to hypoxia led to a significant decrease in survival in the parental cell line, no change in survival was determined in the KO cells (Fig. 5A and KO2 and KO3 Fig. S6A, B). These data were further supported by demonstrating that exposure to hypoxia had a significant impact on proliferation rates when parental cells were returned to normal oxygen levels, whilst those lacking PHGDH were unaffected (Fig. S6C, D). Next, we asked whether the resistance to hypoxia observed in PHGDH KO cells occurred in all media types. When grown in HPLM, cell survival under hypoxia was affected in PHGDH WT but not in KO1 cells (Fig. 5B and KO2 and KO3 Fig. S6E). Exposure to serine-free MEM led to reduced survival rates under hypoxia in both PHGDH Con and KO1 cells (Fig. 5C and KO2 and KO3 Fig. S6F). Together, these data suggest that loss of PHGDH could prime cells for survival in hypoxic conditions. Supportively, the metabolomic analysis showed elevated levels of pyruvate and intracellular lactate (Fig. 5D, E). To further investigate the effect of PHGDH loss on changes in lactate and glycolysis, an extracellular flux glycolytic stress test was carried out. The extracellular acidification rate (ECAR), which can be used as a surrogate for extracellular lactate production, demonstrated a significantly higher rate in the PHGDH KO1 and KO2 cells (Fig. 5F). Furthermore, following the glucose injection, PHGDH-deficient cells (KO1 and KO2) showed a larger increase in ECAR compared to parental controls, indicating a rapid elevation of glycolytic flux (Fig. 5G). To confirm that the resistance to hypoxia observed in the KO cells was PHGDH dependent, a rescue experiment was carried out. Expression of physiological levels of PHGDH in the KO cells restored the sensitivity to hypoxia determined in the parental cell line (Figs. 5H, I and KO2 S6G, H).

### Loss of PHGDH leads to increased HIF-1 activity

Increased lactate has been linked to HIF-1 stability [35–37]. Parental and KO A549 cells were exposed to hypoxia and the speed with which HIF-1α accumulated was determined by western blotting. In response to hypoxia ( < 0.1% O_2_), HIF-1α accumulated faster in the KO cells compared to the parental control cells (Figs. 5J and S7A). To investigate this further, a hypoxia response element (HRE)-luciferase reporter was used to demonstrate that loss of PHGDH led to increased HIF-1 activity under hypoxia in the KO cells (Figs. 5K and KO2 S7B). This was further confirmed by determining the accumulation of known HIF target genes in response to hypoxia in both cell lines (parental and KO). The levels of CAIX, GLUT1, VEGF and LDHA were all found to be significantly higher in the KO1 cells exposed to hypoxia ( < 0.1% O_2_) compared to the parental control (Figs. 5L, M and S7C–F). Together, these data show that loss of PHGDH confers resistance to hypoxic stress, an effect that is reversible by PHGDH re-expression and is associated with enhanced HIF-1α accumulation and transcriptional activity under hypoxia.

## Discussion

Here, we demonstrate that PHGDH loss does not increase radiosensitivity in cancer cells grown in physiologically relevant concentrations of extracellular serine/glycine. Increased radiosensitivity was observed when cells were irradiated in the complete absence of extracellular serine/glycine. Our data support the conclusion that increasing radiosensitivity through a PHGDH-dependent reduction of GSH levels is only achievable in the absence of extracellular serine/glycine.

Importantly, we found that loss of PHGDH increases tolerance to hypoxia in cells cultured in both DMEM and HPLM. Hypoxia is known to drive metabolic reprogramming, including an increased reliance on glycolysis. Elevated extracellular lactate levels have been associated with enhanced tumour invasion and metastasis, as well as impaired immune surveillance in lung, breast and other cancer types [38–41]. Additionally, an increase in lactate has been shown to correlate with radioresistance, both in vitro and in vivo [36–38, 42]. Increased lactate levels have also been shown to stabilise HIF-1α through lactylation, which can block its recognition by the von Hippel–Lindau (VHL) complex [43]. We observed increased glycolysis and ECAR in cells lacking PHGDH, supportive of higher lactate levels, which could therefore contribute to increased HIF-1α accumulation and HIF-1 activity. Moreover, enhanced induction of LDHA in PHGDH-deficient cells in hypoxia is likely to further increase glycolytic flux, thereby promoting lactate production and establishing a positive feedback loop [44]. Overall, these data show that PHGDH loss leads to increased glycolysis, effectively priming them to respond to hypoxic conditions.

Interestingly, we found that PHGDH was induced in a HIF-1-dependent manner in response to <0.1% O_2_ but did not change in milder conditions (2% O_2_), which are also associated with HIF-1 activity. A potential model to explain this expression pattern involves the oxygen-dependency of the hydroxylation pathways, which play key roles in HIF-1α stabilisation and activity. Specifically, inhibition of the prolyl hydroxylases (PHDs) occurs at relatively higher oxygen levels, allowing HIF-1α stabilisation, while Factor Inhibiting HIF (FIH) remains active, restricting recruitment of p300/CBP and limiting transcription [45]. As oxygen levels decrease further, FIH activity is progressively lost, permitting full HIF transcriptional activity and maximal expression of a subset of FIH-sensitive target genes [46]. Furthermore, certain chromatin remodelling proteins, such as KDM6A, are direct oxygen sensors and can change chromatin accessibility and affect HIF binding to the HREs in an oxygen-dependent manner [47]. To fully characterise the mechanism of PHGDH induction in response to hypoxia, chromatin immunoprecipitation of HIF-1 and potentially ATF4 would be required.

In contrast to the data shown here, pharmacological inhibition of PHGDH has also been shown to increase the radiosensitivity of colorectal cancer cells in hypoxic conditions [15]. However, it should be noted that the reported experimental conditions include non-physiological levels of extracellular serine (RPMI, 286 μM serine) [48]; therefore, it is unclear what mechanism underpins the observed increase in radiosensitivity. Moreover, the PHGDH inhibitor used, NCT-503, has been reported to have off-target effects by reducing glucose-derived citrate levels in a PHGDH-independent manner [49]. Inherent biological differences between colorectal and lung cancer cells could also contribute to the differing observations. Similarly, the only other report on PHGDH-dependent radiosensitisation focused on glioblastoma stem cells, which exhibit a distinct metabolic landscape and therapy response [14].

Elevated PHGDH expression has been associated with poor patient outcomes in several studies [50–52]; however, recent evidence suggests a more complex role, as low or heterogeneous PHGDH expression has been linked to cancer cell dissemination and metastatic progression [53]. Taken together, these findings highlight a context-dependent role for PHGDH in tumour biology and suggest that inhibition of serine biosynthesis alone is unlikely to overcome hypoxia-mediated radioresistance in lung cancer. More broadly, this work underscores the importance of considering nutrient availability when evaluating metabolic targets for combination with radiotherapy and cautions against extrapolating radiosensitisation strategies from non-physiological culture conditions to the clinical setting.

## Materials and methods

### Cell culture

Cells were grown in DMEM supplemented with 10% FBS, in a standard humidified incubator at 37 °C and 5% CO_2_. Cell lines used for the study: A549 (human lung adenocarcinoma, ATCC), NCI-H460 (human large cell lung carcinoma, provided by Prof. Geoff Higgins, University of Oxford), RKO (human colorectal cancer cells, ATCC) and RKO^HIF-1α-/-^ [54]. Additional media HPLM (A4899101, Gibco) and MEM (21090022, Gibco). PHGDH knockout clones were generated using a CRISPR-Cas9 ribonucleoprotein-based genome editing approach in accordance with Synthego’s single-cell cloning workflow (guide sequences Table S1). Cells were regularly checked for mycoplasma (Mycoalert mycoplasma detection kit, Lonza).

### Exposure to hypoxia

Cells were incubated in a Whitley A35 chamber (Don Whitley Scientific), Bactron II anaerobic chamber (Sheldon manufacturing) or Whitley H35 Hypoxystation (Don Whitley Scientific). For experiments conducted at <0.1% O_2_ levels, cells were plated on glass dishes. Cells were harvested inside hypoxia chambers with equilibrated solutions.

### Western blotting

Cells were lysed using UTB (9 M urea, 75 mM Tris-HCl, pH 7.5, 0.15 M β-mercaptoethanol) and sonicated briefly. Proteins were separated on a 4–20% polyacrylamide gel (Bio-Rad) and transferred onto a nitrocellulose membrane (Bio-Rad). Odyssey IR imaging technology (LI-COR Biosciences) was used. Primary antibodies: PHGDH (Novus Biologicals, NBP2-46389), HIF-1α (BD Biosciences, 610958), β-actin (Santa Cruz, AC-15), γH2AX (Sigma Aldrich, 05-636).

### RT-qPCR

RNA was prepared using TRIzol (Invitrogen/Life Technologies). cDNA was reverse transcribed from total RNA using the Verso kit (Thermo Scientific). qPCR was performed using SYBR Green PCR Master Mix kit (Applied Biosystems) in a 7500 FAST Real-Time PCR thermocycler with v2.0.5 software (Applied Biosystems). RNA fold change was calculated using a 2^–ΔΔCt^ method in relation to the 18S. The mean of three biological replicates ± SEM is shown. All primer sequences are available (Table S2).

### HIF reporter assay

Cells were transiently transfected with 5x-HRE-luciferase [55] and pCMV-Renilla plasmids using Lipofectamine 2000 transfection reagent (Invitrogen Life Technologies). pCMV-Renilla was used for normalisation purposes. Firefly and Renilla luciferase activities were measured using the Dual Glo Luciferase assay (Promega).

### Irradiation

Cells were irradiated using a Cs-137 irradiator (GSM: GSR D1; dose rate 1.7 Gy min^−1^ at room temperature). For irradiation in hypoxic conditions, cells were sealed in equilibrated air-tight boxes as previously described and transported to the source [56].

### GSH measurements

A probe (FL-1) was used to measure the GSH levels in cells [10]. Cells were seeded (6000/well) in flat-bottomed 96-well plates. As a negative control, cells were treated with N-ethylmaleimide (NEM, 200 μM) for 6 h. FL-1 (10 μM) was then added to each well, and after 1 h, the fluorescence was measured (POLARstar plate reader, BMG LabTech).

### Clonogenic survival assay

Cells were seeded at the appropriate density in 6-well plates. Each treatment condition was performed in triplicate. Colonies were allowed to grow in normal conditions for 10–14 days. Once the colonies had formed ( > 50 cells), the media was removed, and they were stained with crystal violet (0.5% w/v in 50% MeOH and 20% EtOH). Colonies were counted using a manual cell counter (Stuart Scientific). The plating efficiency (PE = colonies counted/cells seeded) and the surviving fraction (SF = PE_IR dose_/PE_0 Gy_) were calculated. A linear-quadratic equation was used for survival data fitting.

### PHGDH overexpression

Knockout cells were transfected with pcDNA3.1 + /C-(K)-DYK expression vector containing the wild-type (WT) PHGDH sequence (GeneScript Clone ID: OHu19607). Transfections were performed using Lipofectamine 2000 (Thermo Fisher Scientific).

### Metabolomics analysis

Cells were fixed and collected in ice-cold methanol. The DNA concentration was normalised to the samples with the lowest concentration (expected range: 50–100 ng/µl). To remove the soluble proteins from the samples, the suspension was passed through a 10 kD molecular weight cut-off filter (Amicon Ultra, Millipore). Metabolomic analysis was carried out as previously described [29].

### Real-time metabolic flux analysis

Real-time analysis of glycolytic stress was performed using the Agilent Seahorse XF analyser. Seahorse XF base medium supplemented with 2 mM Glutamine (GlutaMAX supplement, ThermoFisher Scientific) was used for the experiment. Additionally, the compounds for the glycolytic stress test were prepared: Glucose (10 mM), Oligomycin (1 μM) and 2-Deoxy-D-glucose (50 mM). Three measurement cycles were recorded following the addition of each compound (3 min mix and 3 min measure). Extracellular acidification rate (ECAR) readings were normalised to cell number. For normalisation, cells were fixed with 10% PFA and stained with Hoechst 33342 fluorescent dye (Thermo Fisher Scientific). Cells were counted following the measurement using the Celigo imaging cytometer (Nexcelom). Glycolysis levels were calculated based on the changes in normalised ECAR values following the addition of glucose to the media.

## Supplementary information

Supplementary information

Original data

## Data Availability

The data supporting the results of this study will be made available by the corresponding author upon request. Supplementary information is available at the Cell Death and Discovery website.

## References

[1] Zhou J, Xu Y, Liu J, Feng L, Yu J, Chen D. Global burden of lung cancer in 2022 and projections to 2050: incidence and mortality estimates from GLOBOCAN. Cancer Epidemiol. 2024;93:102693. 39536404 10.1016/j.canep.2024.102693

[2] Kagenaar E, Lugo-Palacios DG, Aggarwal A, Hutchings A, Han L, O’Neill S, et al. Inequalities in the utilisation of curative-intent treatments for early-stage non-small cell lung cancer across urban and rural areas: a population-based study in England. J Cancer Policy. 2025;46:100662. 41213453 10.1016/j.jcpo.2025.100662

[3] Worth KR, Papandreou I, Hammond EM. How the histological structure of some lung cancers shaped almost 70 years of radiobiology. Br J Cancer. 2023;128:407–12. 36344595 10.1038/s41416-022-02041-9 PMC9938174

[4] Skwarski M, Bowler E, Wilson JD, Higgins GS, Hammond EM. Target Tumor Hypoxia. 2020. 10.1007/978-3-030-49701-9_11.

[5] Locasale JW, Grassian AR, Melman T, Lyssiotis CA, Mattaini KR, Bass AJ, et al. Phosphoglycerate dehydrogenase diverts glycolytic flux and contributes to oncogenesis. Nat Genet. 2011;43:869–74. 21804546 10.1038/ng.890 PMC3677549

[6] Possemato R, Marks KM, Shaul YD, Pacold ME, Kim D, Birsoy K, et al. Functional genomics reveal that the serine synthesis pathway is essential in breast cancer. Nature. 2011;476:346–50. 21760589 10.1038/nature10350 PMC3353325

[7] Pikman Y, Ocasio-Martinez N, Alexe G, Dimitrov B, Kitara S, Diehl FF, et al. Targeting serine hydroxymethyltransferases 1 and 2 for T-cell acute lymphoblastic leukemia therapy. Leukemia. 2022;36:348–60. 34341479 10.1038/s41375-021-01361-8 PMC8807390

[8] Renwick SB, Snell K, Baumann U. The crystal structure of human cytosolic serine hydroxymethyltransferase: a target for cancer chemotherapy. Structure. 1998;6:1105–16. 9753690 10.1016/s0969-2126(98)00112-9

[9] Hacker L, Sarsam E, Conway SJ, Hammond EM. A guide to reactive oxygen species in tumour hypoxia: measurement and therapeutic implications. Mol Oncol. 2025;19:3003–22. 41158026 10.1002/1878-0261.70151 PMC12591319

[10] Twigger SA, Dominguez B, Porto V, Hacker L, Chalmers AJ, Breckenridge R, et al. The activity of therapeutic molecular cluster Ag5 is dependent on oxygen level and HIF-1- mediated signalling. Redox Biol. 2024;76:103326. 39180984 10.1016/j.redox.2024.103326 PMC11388176

[11] Dong J-K, Lei H-M, Liang Q, Tang Y-B, Zhou Y, Wang Y, et al. Overcoming erlotinib resistance in EGFR mutation-positive lung adenocarcinomas through repression of phosphoglycerate dehydrogenase. Theranostics. 2018;8:1808–23. 29556358 10.7150/thno.23177 PMC5858502

[12] Zhang X, Bai W. Repression of phosphoglycerate dehydrogenase sensitizes triple-negative breast cancer to doxorubicin. Cancer Chemother Pharm. 2016;78:655–9. 10.1007/s00280-016-3117-4 27473325

[13] Mancini C, Lori G, Mattei G, Iozzo M, Desideri D, Cianchi F, et al. PHGDH drives 5-FU chemoresistance in colorectal cancer through the Hedgehog signaling. J Exp Clin Cancer Res. 2025;44:198. 40640948 10.1186/s13046-025-03447-y PMC12243184

[14] Liu X, Liu B, Wang J, Liu H, Wu J, Qi Y, et al. PHGDH activation fuels glioblastoma progression and radioresistance via serine synthesis pathway. J Exp Clin Cancer Res. 2025;44:99. 40102981 10.1186/s13046-025-03361-3 PMC11921657

[15] Van de Gucht M, Dufait I, Kerkhove L, Corbet C, de Mey S, Jiang H, et al. Inhibition of phosphoglycerate dehydrogenase radiosensitizes human colorectal cancer cells under hypoxic conditions. Cancers. 2022;14:5060. 36291844 10.3390/cancers14205060 PMC9599856

[16] Sánchez-Castillo A, Heylen E, Hounjet J, Savelkouls KG, Lieuwes NG, Biemans R, et al. Targeting serine/glycine metabolism improves radiotherapy response in non-small cell lung cancer. Br J Cancer. 2024;130:568–84. 38160212 10.1038/s41416-023-02553-y PMC10876524

[17] Falcone M, Uribe AH, Papalazarou V, Newman AC, Athineos D, Stevenson K, et al. Sensitisation of cancer cells to radiotherapy by serine and glycine starvation. Br J Cancer. 2022;127:1773–86. 36115879 10.1038/s41416-022-01965-6 PMC9643498

[18] Yang Y, Li S, Yang Y, Li Q, Liu Y, Cao J. ATF4/PHGDH mediates the effects of ER stress on cadmium-induced autophagy and glycolysis. Toxicology. 2024;509:153976. 39427783 10.1016/j.tox.2024.153976

[19] Nagelkerke A, Bussink J, Van Der Kogel AJ, Sweep FCGJ, Span PN. The PERK/ATF4/LAMP3-arm of the unfolded protein response affects radioresistance by interfering with the DNA damage response. Radiother Oncol. 2013;108:415–21. 23891100 10.1016/j.radonc.2013.06.037

[20] Rzymski T, Milani M, Pike L, Buffa F, Mellor HR, Winchester L, et al. Regulation of autophagy by ATF4 in response to severe hypoxia. Oncogene. 2010;29:4424–35. 20514020 10.1038/onc.2010.191

[21] Comprehensive genomic characterization of squamous cell lung cancers. Nature 2012;489:519–25. 10.1038/nature11404 PMC3466113 22960745

[22] Comprehensive molecular profiling of lung adenocarcinoma. Nature 2014; 511: 543–50. 10.1038/nature13385 PMC4231481 25079552

[23] Winter SC, Buffa FM, Silva P, Miller C, Valentine HR, Turley H, et al. Relation of a hypoxia metagene derived from head and neck cancer to prognosis of multiple cancers. Cancer Res. 2007;67:3441–9. 17409455 10.1158/0008-5472.CAN-06-3322

[24] Buffa FM, Harris AL, West CM, Miller CJ. Large meta-analysis of multiple cancers reveals a common, compact and highly prognostic hypoxia metagene. Br J Cancer. 2010;102:428–35. 20087356 10.1038/sj.bjc.6605450 PMC2816644

[25] Wykoff CC, Beasley N, Watson PH, Campo L, Chia SK, English R, et al. Expression of the hypoxia-inducible and tumor-associated carbonic anhydrases in ductal carcinoma in situ of the breast. Am J Pathol. 2001;158:1011–9. 11238049 10.1016/S0002-9440(10)64048-5 PMC1850356

[26] Zhang D, Li AM, Hu G, Huang M, Yang F, Zhang L, et al. PHGDH-mediated endothelial metabolism drives glioblastoma resistance to chimeric antigen receptor T cell immunotherapy. Cell Metab. 2023;35:517–534.e8. 36804058 10.1016/j.cmet.2023.01.010 PMC10088869

[27] Dai Z, Chen L, Pan K, Zhao X, Xu W, Du J, et al. Multi-omics analysis of the role of PHGDH in colon cancer. Technol Cancer Res Treat. 2023;22:15330338221145994. 36707056 10.1177/15330338221145994 PMC9896097

[28] Bartoszewska S, Collawn JF. Unfolded protein response (UPR) integrated signaling networks determine cell fate during hypoxia. Cell Mol Biol Lett. 2020;25:18. 32190062 10.1186/s11658-020-00212-1 PMC7071609

[29] Williams R, Walsby-Tickle J, Hvinden IC, Legge I, Kacerova T, Lee KV, et al. Metabolomics using anion-exchange chromatography mass spectrometry for the analysis of cells, tissues and biofluids. Nat Protoc. 2025. 10.1038/s41596-025-01222-z. 40847241 10.1038/s41596-025-01222-z

[30] Walsby-Tickle J, Gannon J, Hvinden I, Bardella C, Abboud MI, Nazeer A, et al. Anion-exchange chromatography mass spectrometry provides extensive coverage of primary metabolic pathways revealing altered metabolism in IDH1 mutant cells. Commun Biol. 2020;3:247. 32433536 10.1038/s42003-020-0957-6 PMC7239943

[31] Papalazarou V, Newman AC, Huerta-Uribe A, Legrave NM, Falcone M, Zhang T, et al. Phenotypic profiling of solute carriers characterizes serine transport in cancer. Nat Metab. 2023;5:2148–68. 38066114 10.1038/s42255-023-00936-2 PMC10730406

[32] Psychogios N, Hau DD, Peng J, Guo AC, Mandal R, Bouatra S, et al. The human serum metabolome. PLoS One. 2011;6:e16957. 21359215 10.1371/journal.pone.0016957 PMC3040193

[33] Hennequart M, Labuschagne CF, Tajan M, Pilley SE, Cheung EC, Legrave NM, et al. The impact of physiological metabolite levels on serine uptake, synthesis and utilization in cancer cells. Nat Commun. 2021;12:6176. 34702840 10.1038/s41467-021-26395-5 PMC8548528

[34] Dulbecco R, Freeman G. Plaque production by the polyoma virus. Virology. 1959;8:396–7. 13669362 10.1016/0042-6822(59)90043-1

[35] De Saedeleer CJ, Copetti T, Porporato PE, Verrax J, Feron O, Sonveaux P. Lactate activates HIF-1 in oxidative but not in Warburg-phenotype human tumor cells. PLoS One. 2012;7:e46571. 23082126 10.1371/journal.pone.0046571 PMC3474765

[36] Gao J, Liu R, Huang K, Li Z, Sheng X, Chakraborty K, et al. Dynamic investigation of hypoxia-induced L-lactylation. Proc. Natl. Acad. Sci. 2025;122. 10.1073/pnas.2404899122. 10.1073/pnas.2404899122 PMC11912421 40030031

[37] Kshitiz, Afzal J, Suhail Y, Chang H, Hubbi ME, Hamidzadeh A, et al. Lactate-dependent chaperone-mediated autophagy induces oscillatory HIF-1α activity promoting proliferation of hypoxic cells. Cell Syst. 2022;13:1048–64.e7. 36462504 10.1016/j.cels.2022.11.003 PMC10012408

[38] Xie H, Hanai J, Ren J-G, Kats L, Burgess K, Bhargava P, et al. Targeting lactate dehydrogenase-A inhibits tumorigenesis and tumor progression in mouse models of lung cancer and impacts tumor-initiating cells. Cell Metab. 2014;19:795–809. 24726384 10.1016/j.cmet.2014.03.003 PMC4096909

[39] Brand A, Singer K, Koehl GE, Kolitzus M, Schoenhammer G, Thiel A, et al. LDHA-associated lactic acid production blunts tumor immunosurveillance by T and NK cells. Cell Metab. 2016;24:657–71. 27641098 10.1016/j.cmet.2016.08.011

[40] Faubert B, Li KY, Cai L, Hensley CT, Kim J, Zacharias LG, et al. Lactate metabolism in human lung tumors. Cell. 2017;171:358–371.e9. 28985563 10.1016/j.cell.2017.09.019 PMC5684706

[41] Brizel DM, Schroeder T, Scher RL, Walenta S, Clough RW, Dewhirst MW, et al. Elevated tumor lactate concentrations predict for an increased risk of metastases in head-and-neck cancer. Int J Radiat Oncol Biol Phys. 2001;51:349–53. 11567808 10.1016/s0360-3016(01)01630-3

[42] Doherty M, Feng J, Wang T, Yin C, Byrne NM, Chambers S, et al. Metabolic radiosensitization by targeting lactate metabolism with microfluidic liposomal nanocarriers. ACS Biomater Sci Eng. 2026. 10.1021/acsbiomaterials.5c02175. 41603671 10.1021/acsbiomaterials.5c02175 PMC12892243

[43] Li C, Fu C, Zhou W, Li H, Liu Z, Wu G, et al. Lactylation modification of HIF-1α enhances its stability by blocking VHL recognition. Cell Commun Signal. 2025;23:364. 40760493 10.1186/s12964-025-02366-x PMC12323271

[44] Semenza GL, Jiang B-H, Leung SW, Passantino R, Concordet J-P, Maire P, et al. Hypoxia response elements in the aldolase A, enolase 1, and lactate dehydrogenase A gene promoters contain essential binding sites for hypoxia-inducible factor 1. J Biol Chem. 1996;271:32529–37. 8955077 10.1074/jbc.271.51.32529

[45] Tarhonskaya H, Hardy AP, Howe EA, Loik ND, Kramer HB, McCullagh JSO, et al. Kinetic investigations of the role of factor inhibiting hypoxia-inducible factor (FIH) as an oxygen sensor. J Biol Chem. 2015;290:19726–42. 26112411 10.1074/jbc.M115.653014 PMC4528135

[46] Dayan F, Roux D, Brahimi-Horn MC, Pouyssegur J, Mazure NM. The oxygen sensor factor-inhibiting hypoxia-inducible factor-1 controls expression of distinct genes through the bifunctional transcriptional character of hypoxia-inducible factor-1α. Cancer Res. 2006;66:3688–98. 16585195 10.1158/0008-5472.CAN-05-4564

[47] Chakraborty AA, Laukka T, Myllykoski M, Ringel AE, Booker MA, Tolstorukov MY, et al. Histone demethylase KDM6A directly senses oxygen to control chromatin and cell fate. Science. 2019;363:1217–22. 30872525 10.1126/science.aaw1026 PMC7336390

[48] Moore GE, Gerner RE, Franklin HA. Culture of normal human leukocytes. JAMA. 1967;199:519–24. 4960081

[49] Arlt B, Mastrobuoni G, Wuenschel J, Astrahantseff K, Eggert A, Kempa S, et al. Inhibiting PHGDH with NCT-503 reroutes glucose-derived carbons into the TCA cycle, independently of its on-target effect. J Enzym Inhib Med Chem. 2021;36:1282–9. 10.1080/14756366.2021.1935917 PMC8253182 34192988

[50] Metcalf S, Petri BJ, Kruer T, Green B, Dougherty S, Wittliff JL, et al. Serine synthesis influences tamoxifen response in ER+ human breast carcinoma. Endocr Relat Cancer. 2021;28:27–37. 33112838 10.1530/ERC-19-0510 PMC7780089

[51] Itoyama R, Yasuda-Yoshihara N, Kitamura F, Yasuda T, Bu L, Yonemura A, et al. Metabolic shift to serine biosynthesis through 3-PG accumulation and PHGDH induction promotes tumor growth in pancreatic cancer. Cancer Lett. 2021;523:29–42. 34508795 10.1016/j.canlet.2021.09.007

[52] Jia XQ, Zhang S, Zhu HJ, Wang W, Zhu JH, Wang XD, et al. Increased expression of PHGDH and prognostic significance in colorectal cancer. Transl Oncol. 2016;9:191–6. 27267836 10.1016/j.tranon.2016.03.006 PMC4907894

[53] Rossi M, Altea-Manzano P, Demicco M, Doglioni G, Bornes L, Fukano M, et al. PHGDH heterogeneity potentiates cancer cell dissemination and metastasis. Nature. 2022;605:747–53. 35585241 10.1038/s41586-022-04758-2 PMC9888363

[54] Dang DT, Chen F, Gardner LB, Cummins JM, Rago C, Bunz F, et al. Hypoxia-inducible factor-1α promotes nonhypoxia-mediated proliferation in colon cancer cells and xenografts. Cancer Res. 2006;66:1684–93. 16452228 10.1158/0008-5472.CAN-05-2887

[55] Shibata T, Giaccia AJ, Brown JM. Development of a hypoxia-responsive vector for tumor-specific gene therapy. Gene Ther. 2000;7:493–8. 10757022 10.1038/sj.gt.3301124

[56] Anbalagan S, Pires IM, Blick C, Hill MA, Ferguson DJP, Chan DA, et al. Radiosensitization of renal cell carcinoma in vitro through the induction of autophagy. Radiother Oncol. 2012;103:388–93. 22551566 10.1016/j.radonc.2012.04.001

[57] Wishart DS, Jewison T, Guo AC, Wilson M, Knox C, Liu Y, et al. HMDB 3.0—the human metabolome database in 2013. Nucleic Acids Res. 2012;41:D801–D807. 23161693 10.1093/nar/gks1065 PMC3531200

