# Supplementary material for: PHGDH loss promotes hypoxia tolerance through glycolytic reprogramming and enhanced HIF-1 activity
Source: Cell Death Discov. 2026 Jun 10;12:335. doi: 10.1038/s41420-026-03194-9 (PMC13478179; doi:10.1038/s41420-026-03194-9)

Uncropped western blots for Petrosyan et al.

Figure 1B

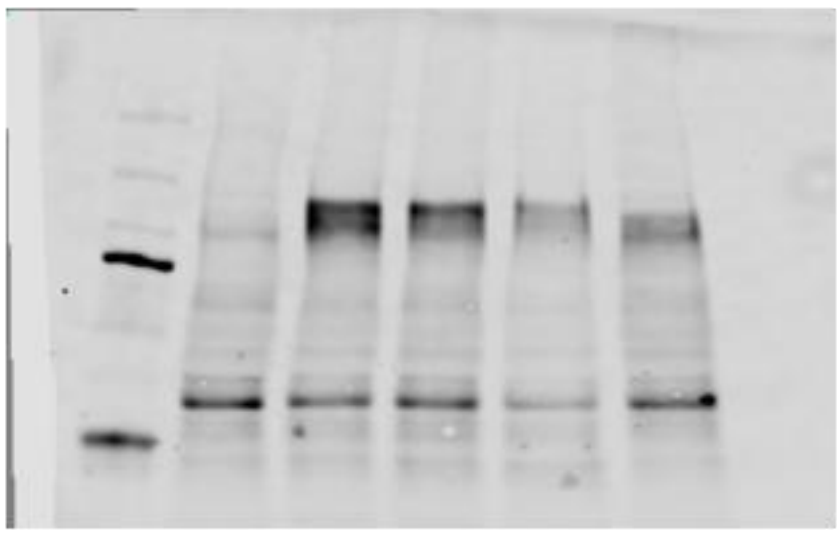

HIF-1α

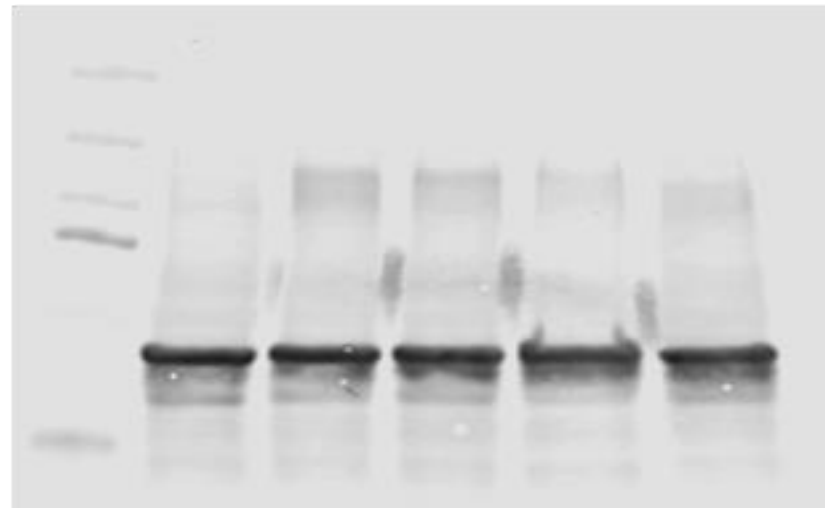

β-actin

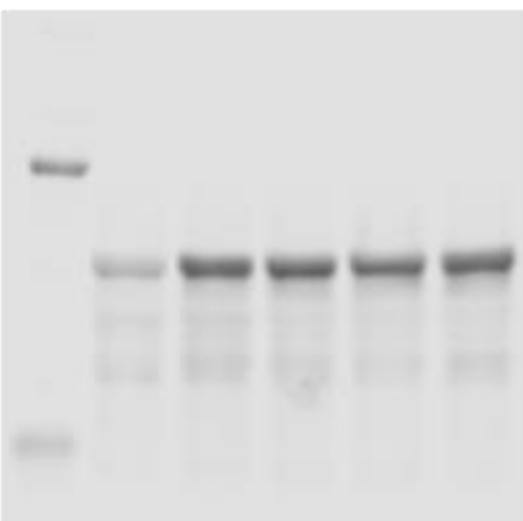

PHGDH

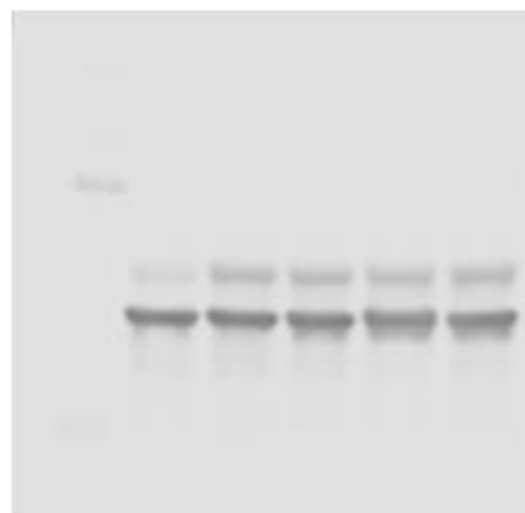

β-actin

Figure 2B

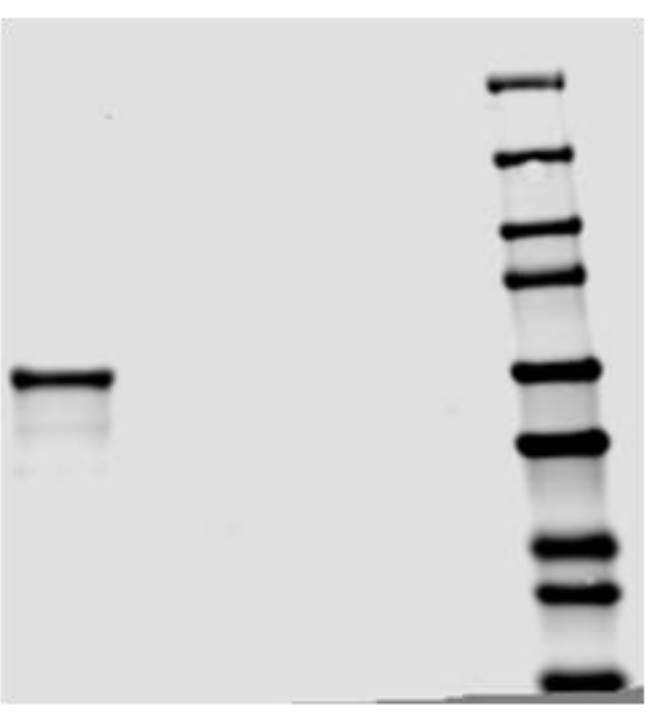

PHGDH

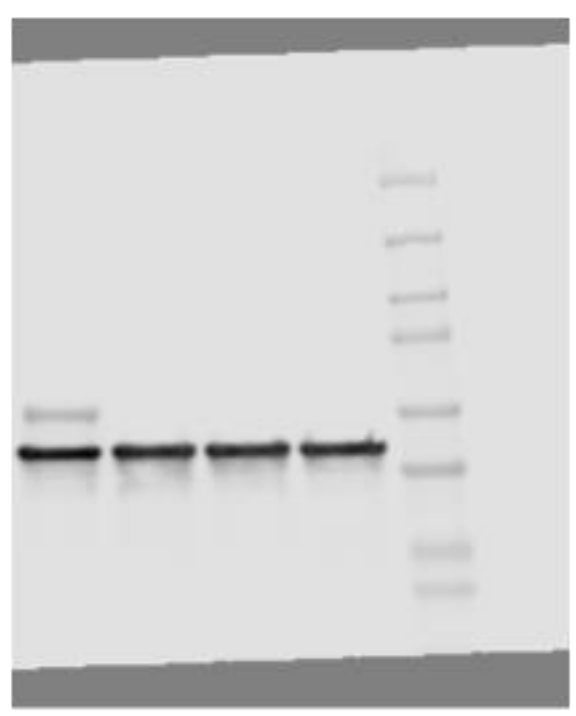

β-actin

Figure 4E-G

Figure 4E

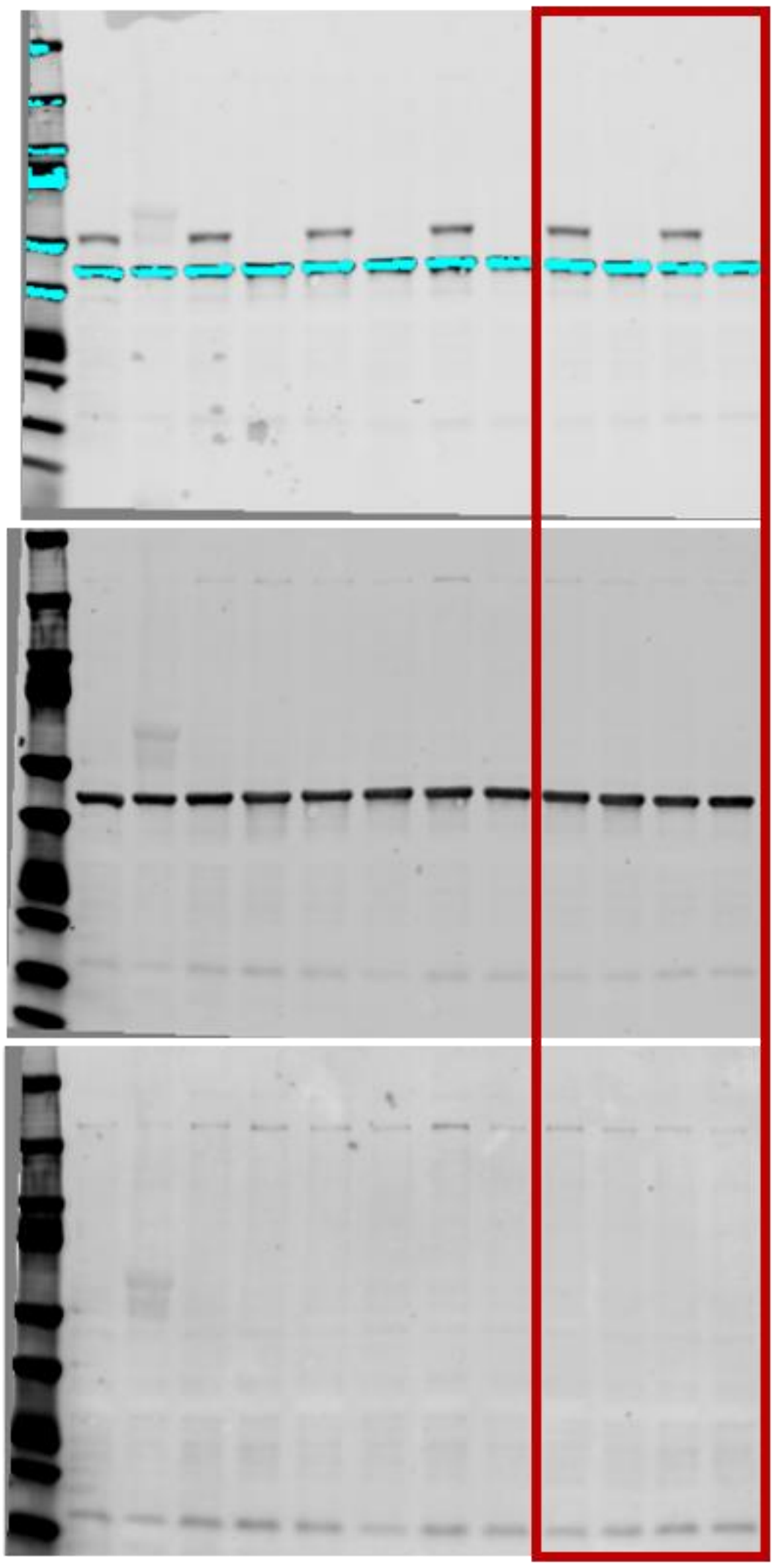

Figure 4F

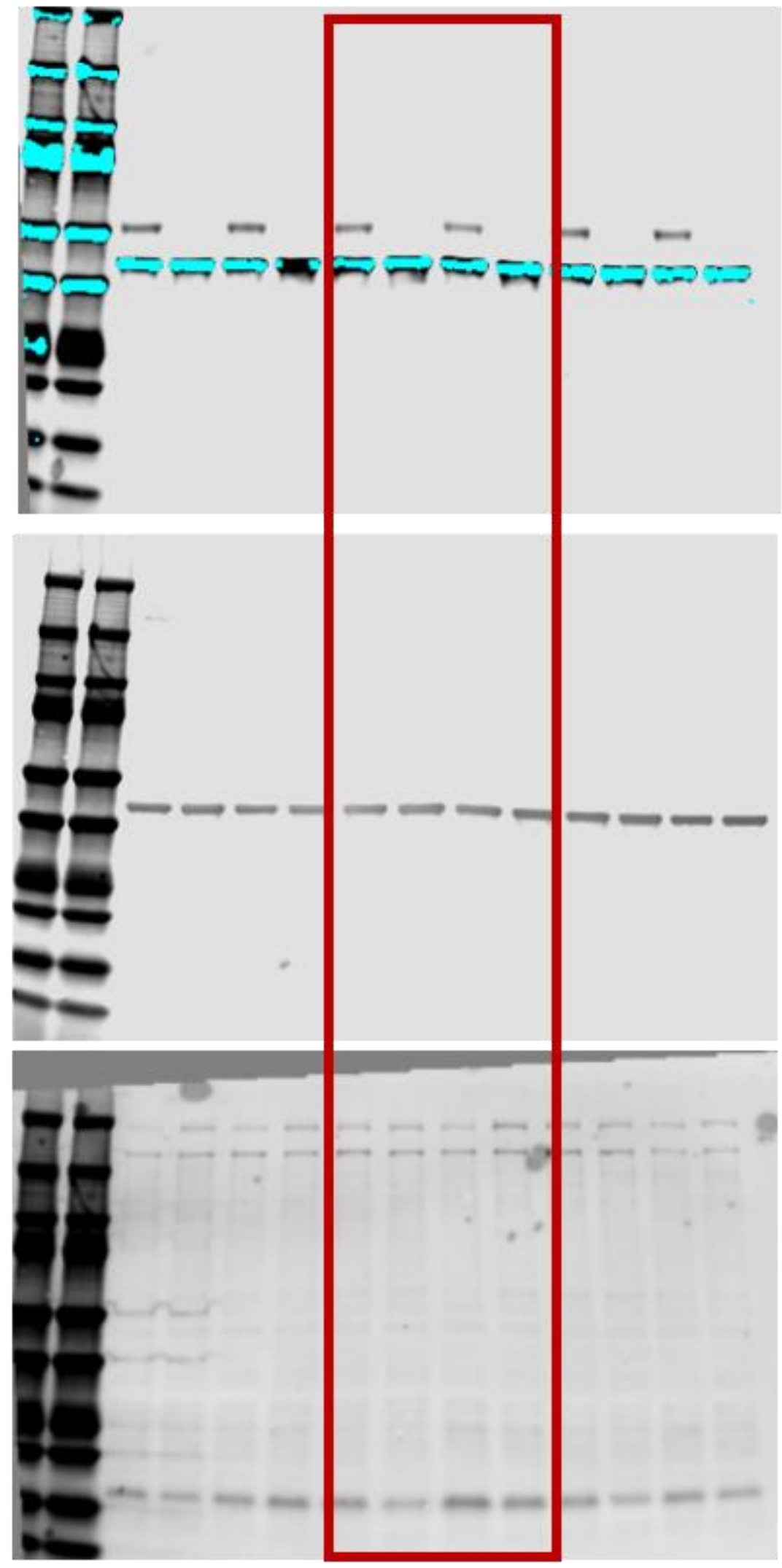

Figure 4G

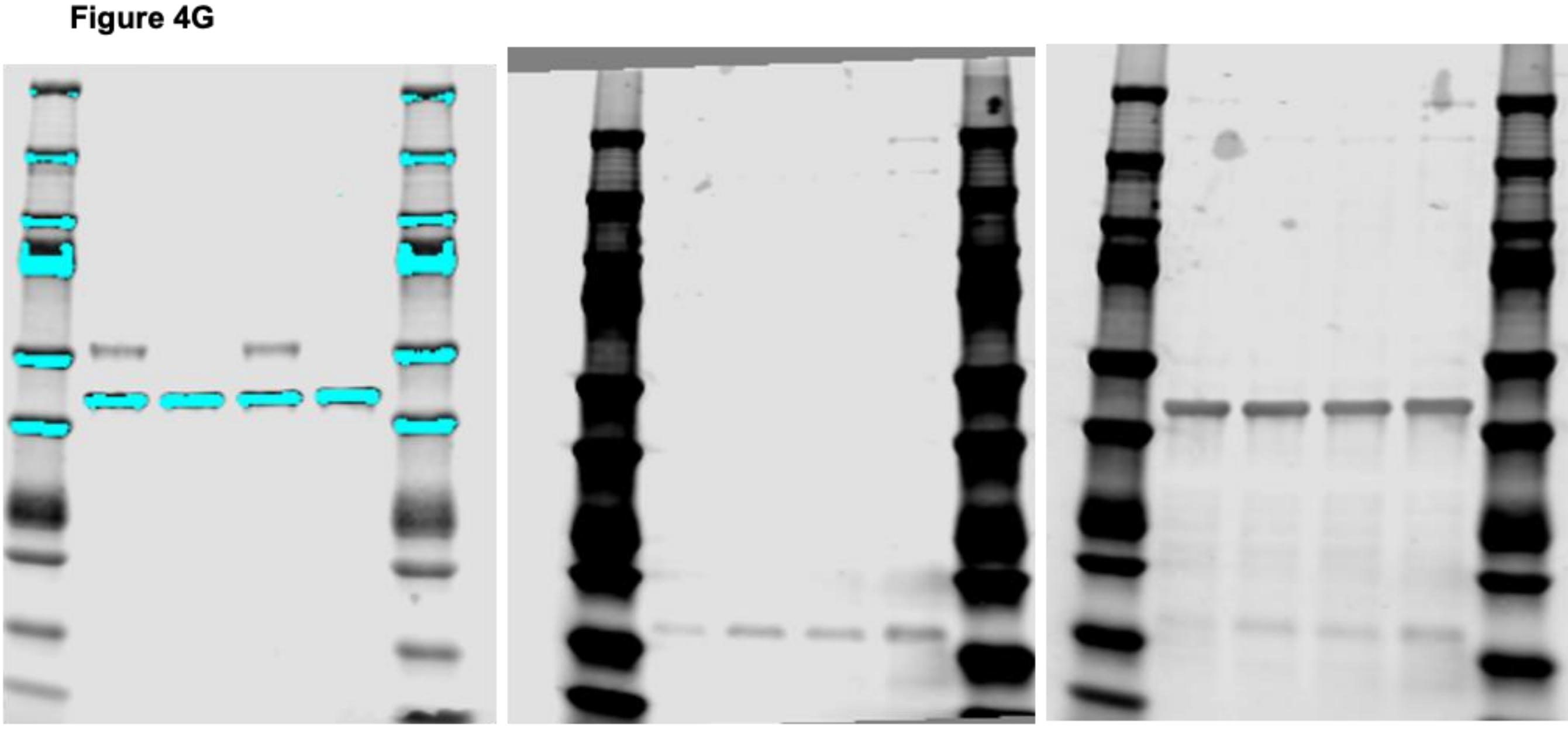

Figure 5I and S6H

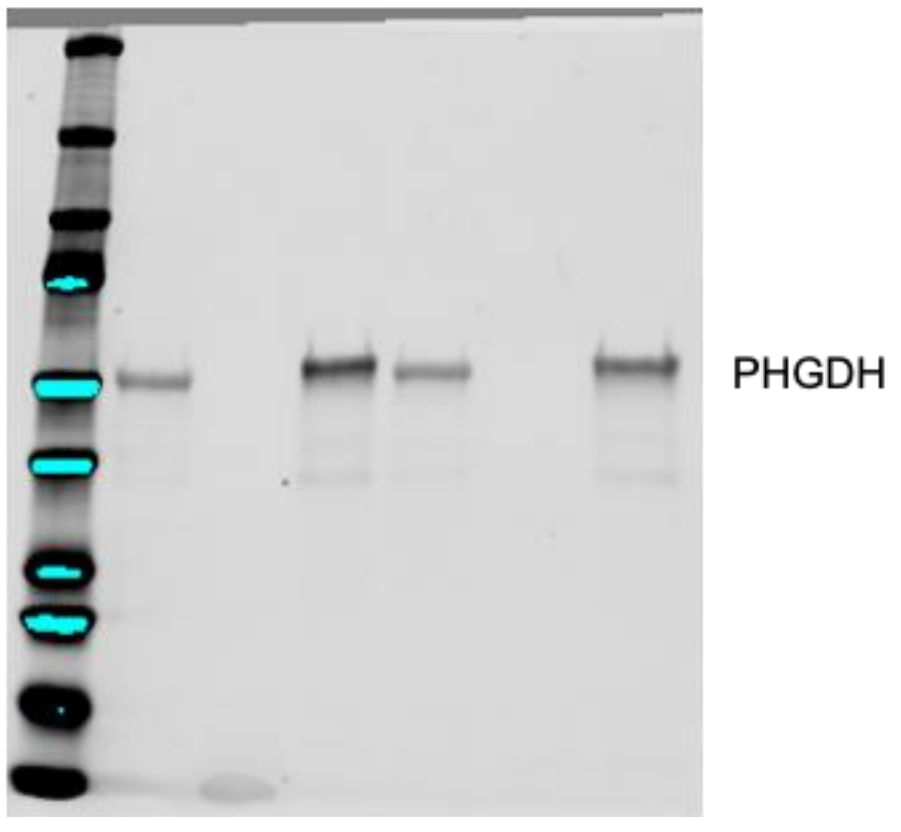

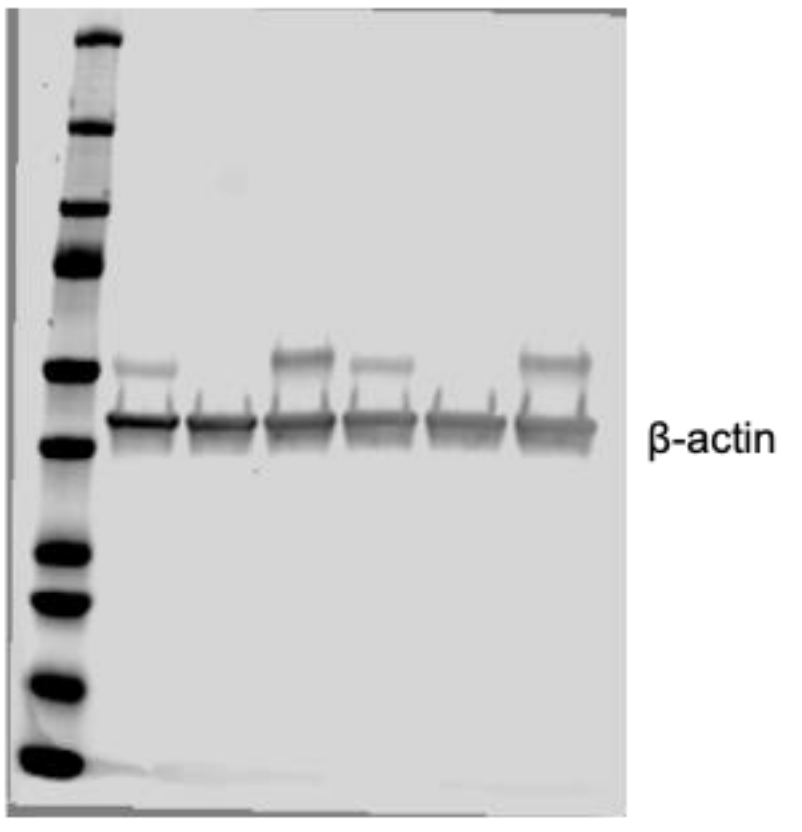

Figure S2D

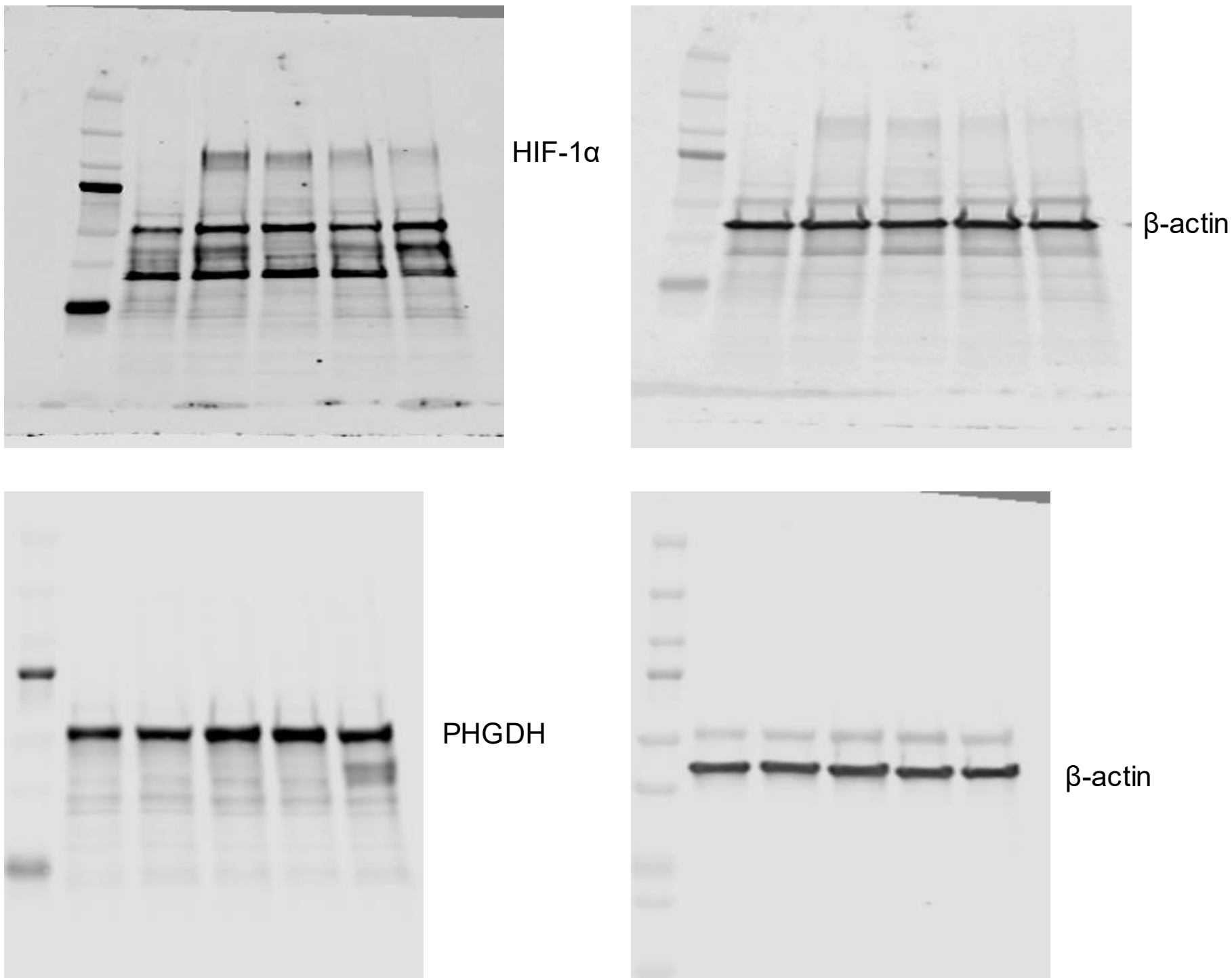

Figure S3G, S3H

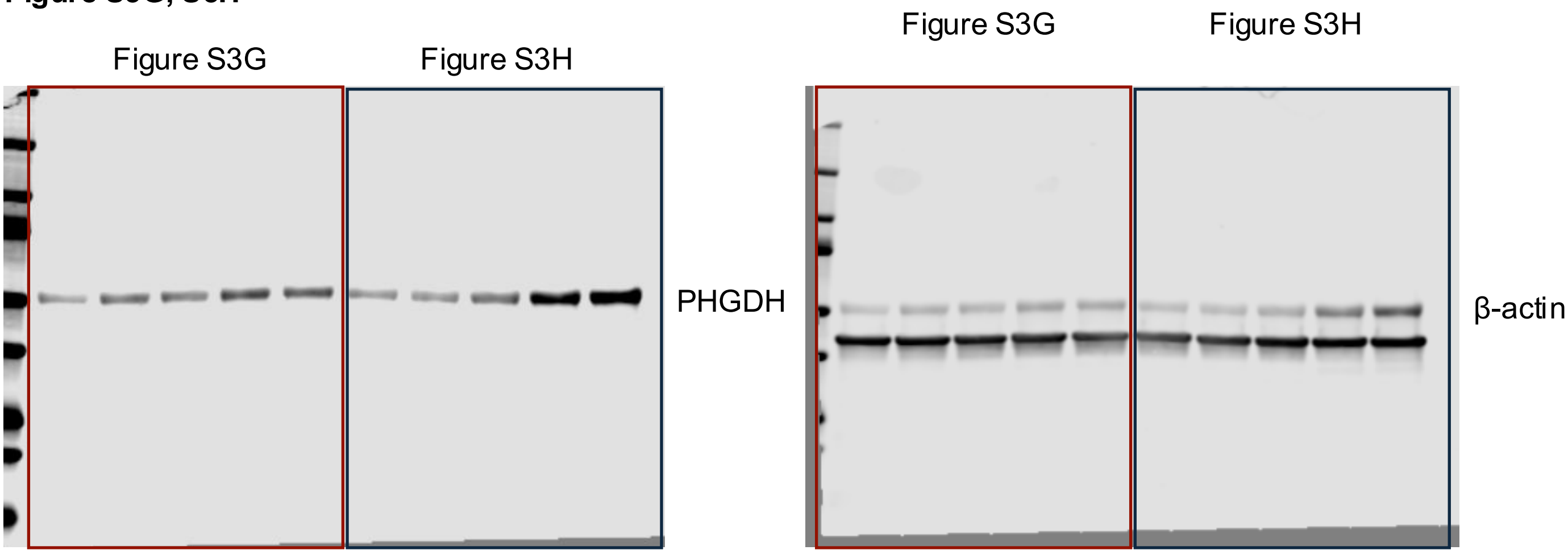

Figure S3I

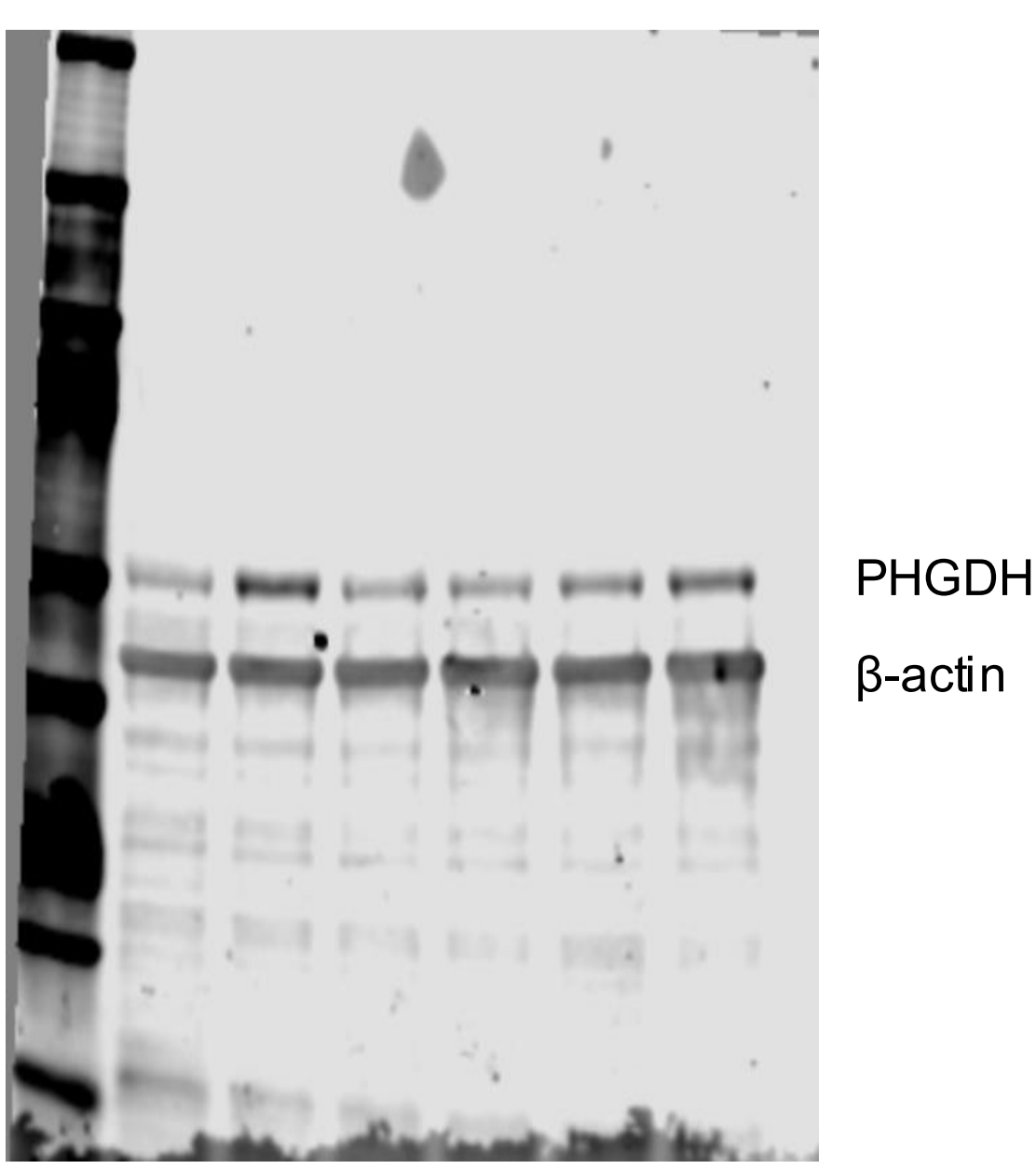

Figure S5B

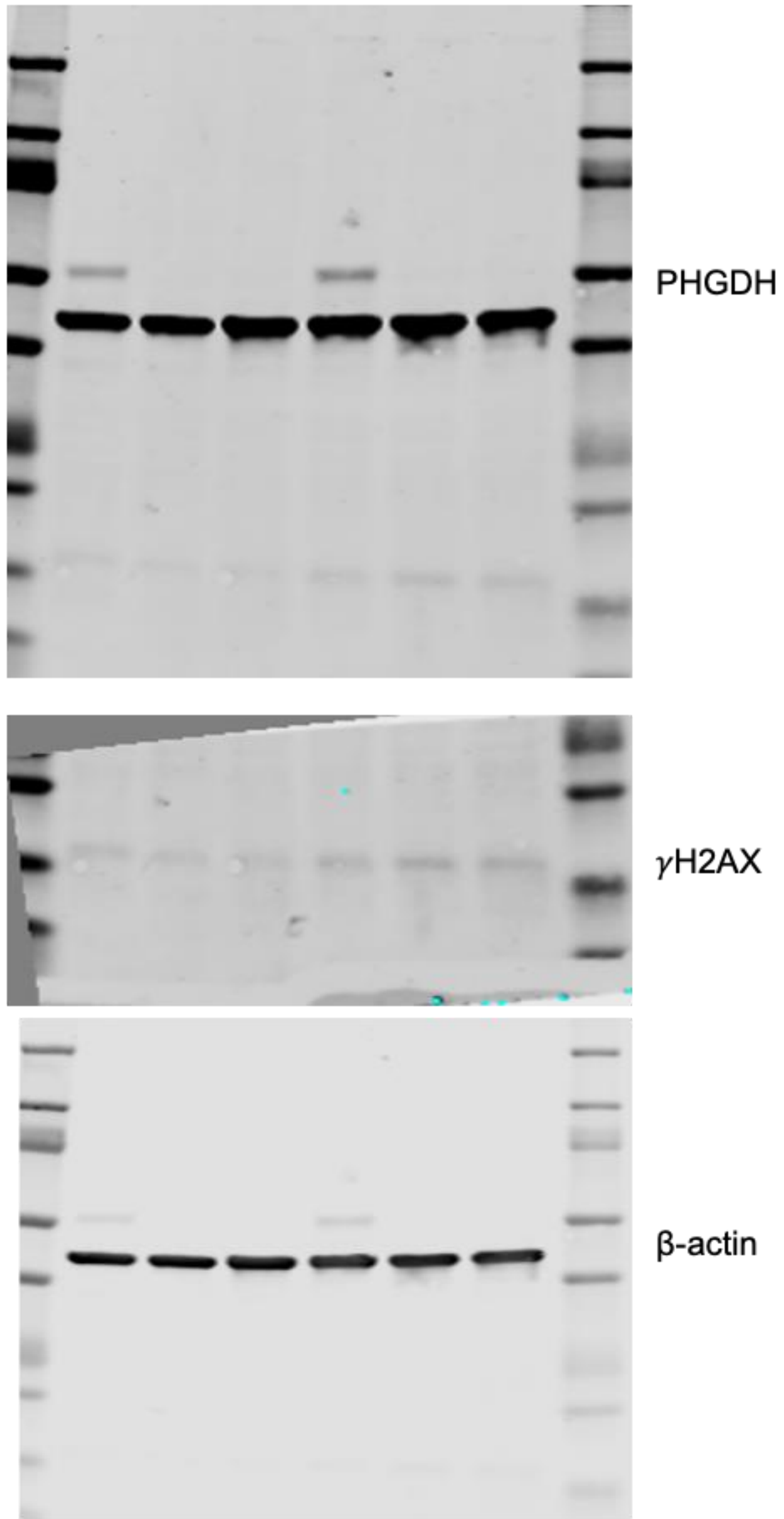

Figure S5C

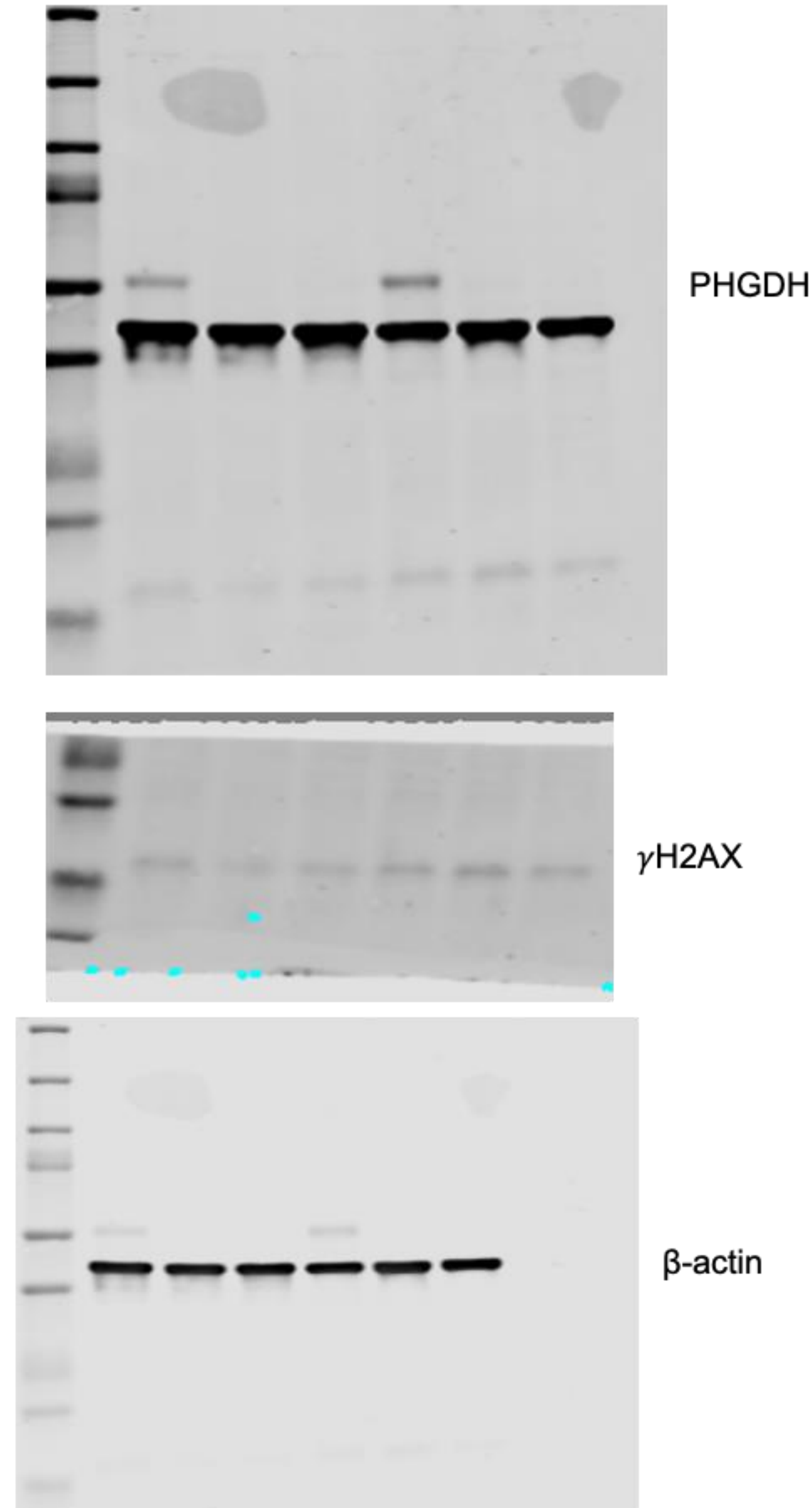

Figure S5D

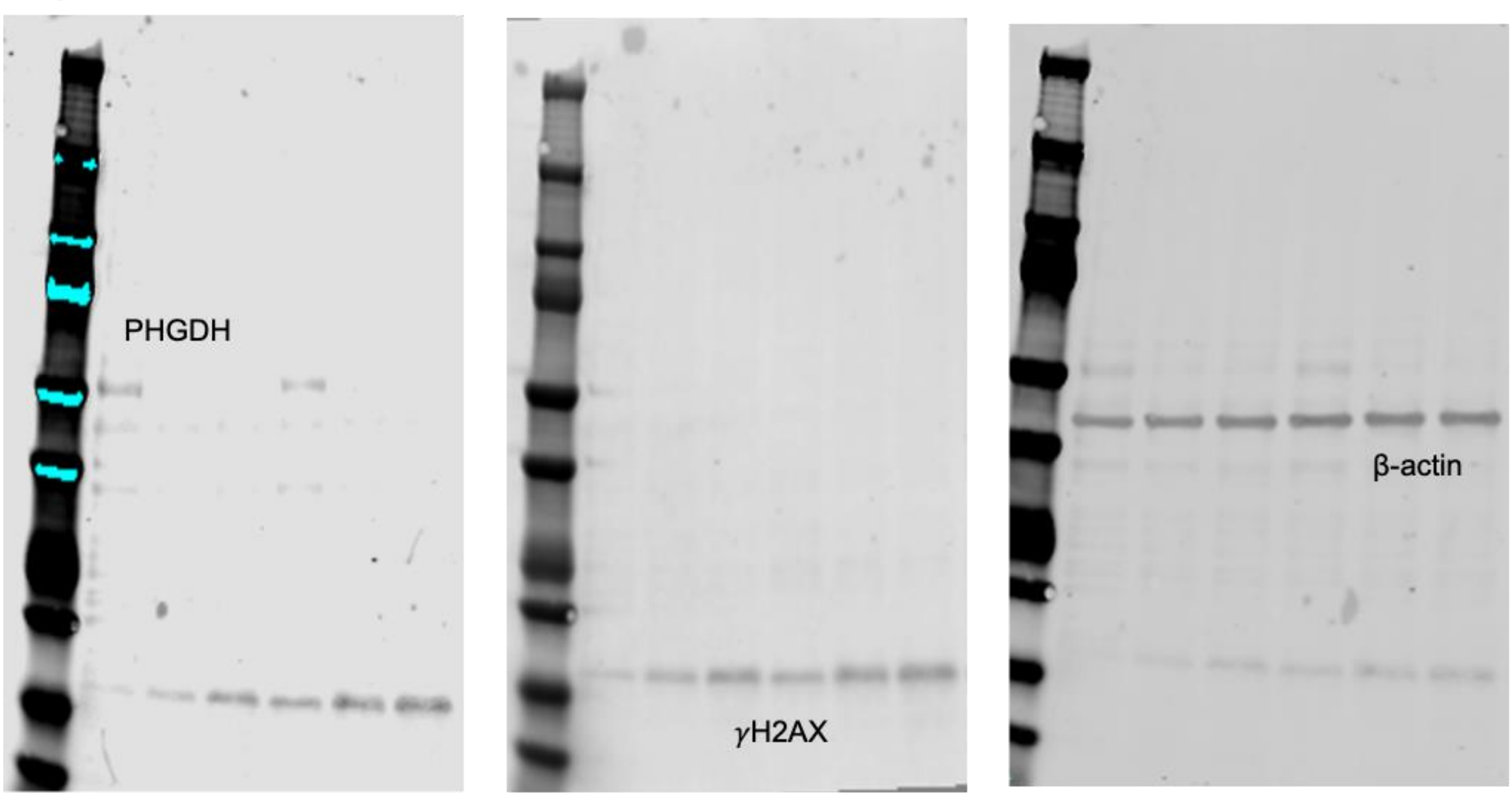

Figure S6B

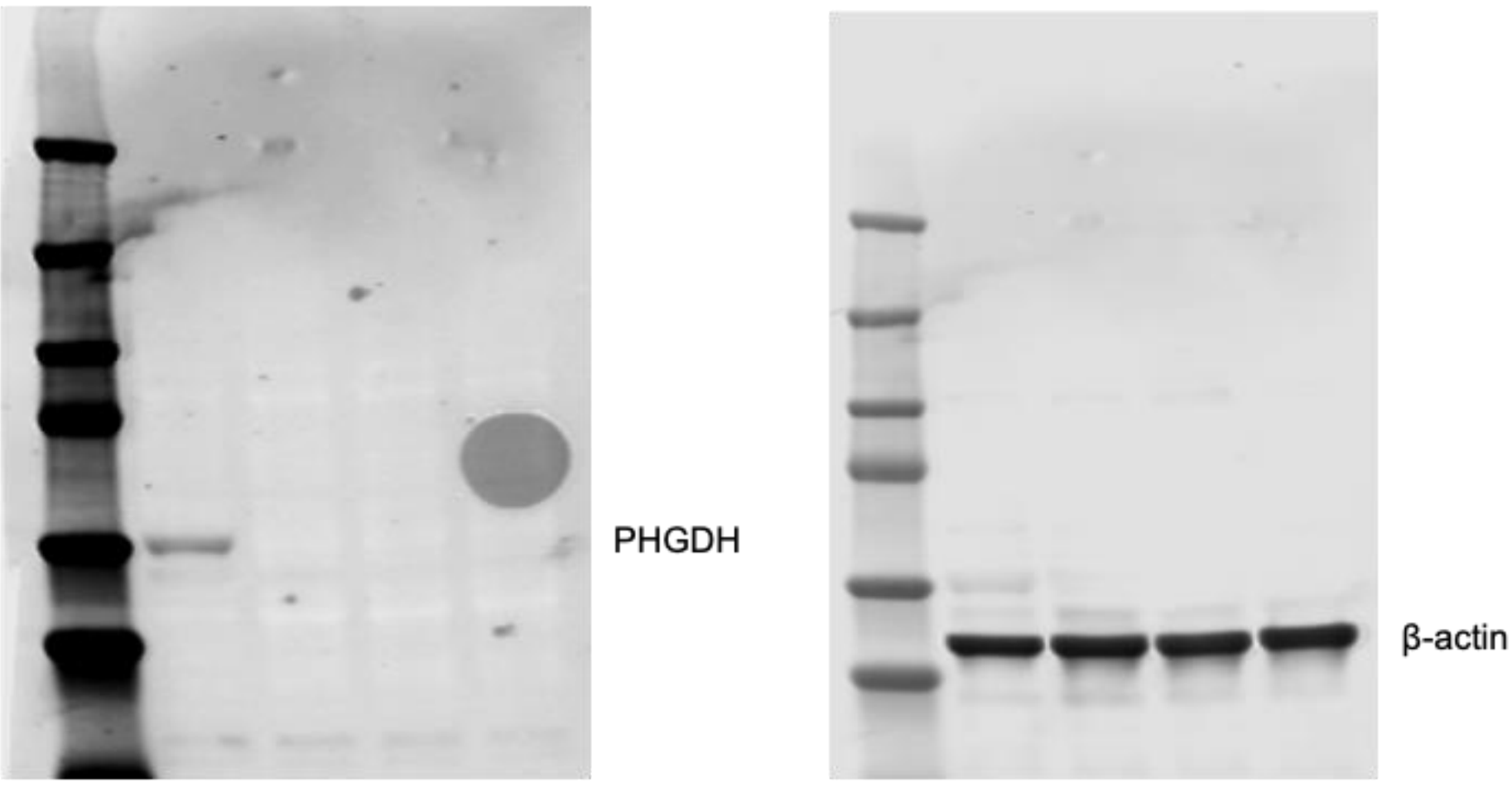

Figure S7A

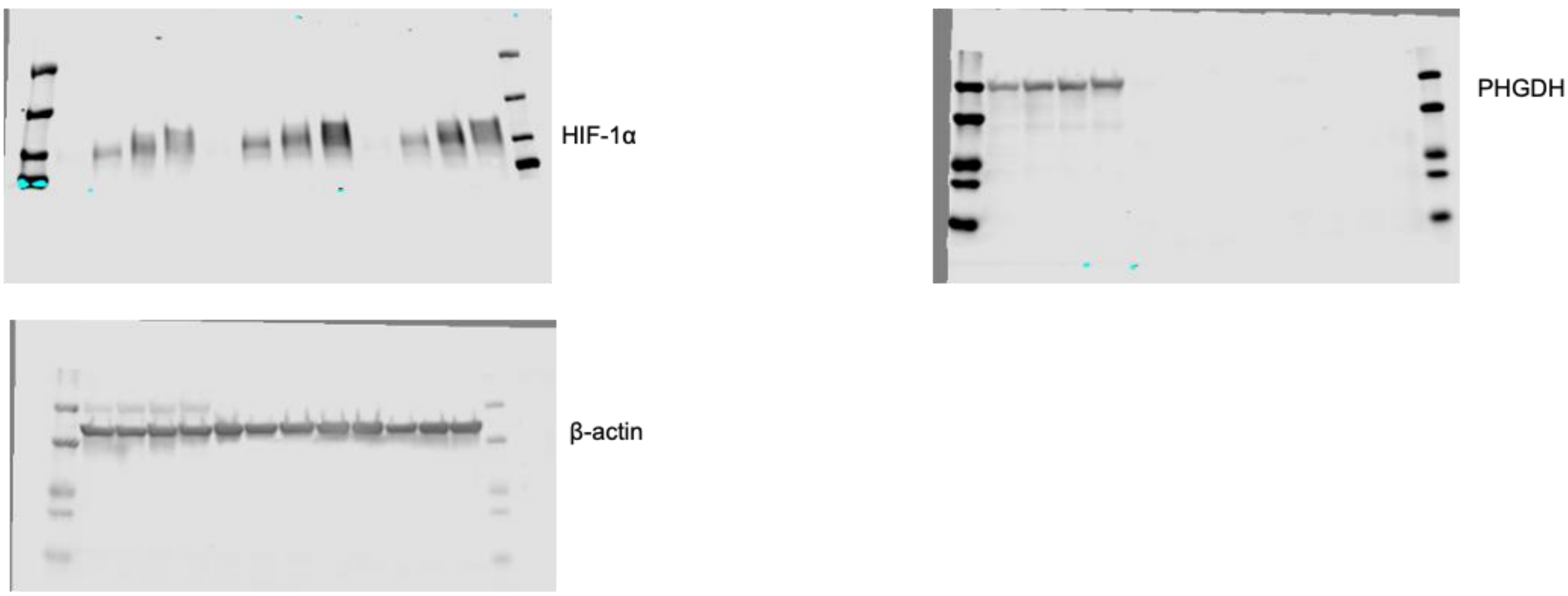

Supplement: Supplementary file 1 [file 41420_2026_3194_MOESM2_ESM.pdf]
