# Supplementary material for: PHGDH loss promotes hypoxia tolerance through glycolytic reprogramming and enhanced HIF-1 activity
Source: Cell Death Discov. 2026 Jun 10;12:335. doi: 10.1038/s41420-026-03194-9 (PMC13478179; doi:10.1038/s41420-026-03194-9)
Supplement: Supplementary file 2 [file 41420_2026_3194_MOESM1_ESM.docx]

**PHGDH loss promotes hypoxia tolerance through glycolytic reprogramming and enhanced HIF-1 activity**

Eduard Petrosyan^1^, Louise A. W. Martin^1^, Isabelle Legge^2^, John Walsby-Tickle^2^, Adam C. Sedgwick^3^, Mark A. Hill^1^, James S. O. McCullagh^2^, Monica M. Olcina^1^, Ester M. Hammond^1^*

^1^Department of Oncology, The University of Oxford, Oxford, OX3 7DQ, UK ^2^Chemistry Research Laboratory, Department of Chemistry, University of Oxford, Mansfield Road, Oxford, OX1 3TA, UK ^3^Department of Chemistry, King's College London, London, SE1 1DB, UK

*Corresponding author **-** ester.hammond@oncology.ox.ac.uk

EMH – ORCID – 0000-0002-2335-3146

EP – ORCID – 0000-0002-8533-9331

**Contents**

SI methods	page 2

SI Tables	page 2

SI Figures	page 4

**SI Methods**

OER calculation

The oxygen enhancement ratio (OER) was defined as the radiation dose ratio between hypoxic and normoxic conditions at 37% survival.

**SI Tables**

**Table S1. Guide RNA (gRNA) sequences used for generating PHDGH CRISPR-Cas9 KO**

| Guide # | Sequence | Exon |
| --- | --- | --- |
| 1 | U*U*U*CUGCUUCAGGACUGUGA | 2 |
| 2 | A*G*C*UGCGUUGAUGACAUCAG | 2 |
| 3 | A*G*G*UGUGGACAAUGUGGAUC | 2 |

**Table S2** **– Primer sequences for RT-qPCR.** Sequences for forward (F) and reverse (R) primers are shown.

| mRNA | Sequence |
| --- | --- |
| 18S | F- GCCCGAAGCGTTTACTTTGA  R- TCCATTATTCCTAGCTGCGGTATC |
| PHGDH | F- CTGCGGAAAGTGCTCATCAGT  R- TGGCAGAGCGAACAATAAGGC |
| CAIX | F- CTTGGAAGAAATCGCTGAGG  R- TGGAAGTAGCGGCTGAAGTC |
| ATF4 | F- TGACCTGGAAACCATGCCAG  R- AATGATCTGGAGTGGAGGAC |
| GLUT1 | F- TTGCAGGCTTCTCCAACTGGAC  R- CAGAACCAGGAGCACAGTGAAG |
| VEGF | F- ATCTTCAAGCCATCCTGTGTGC  R- CAAGGCCCACAGGGATTTTC |
| LDHA | F- AGGCTACACATCCTGGGCTAT  R- CCCAAAATGCAAGGAACACTA |

**Table S3** - **Media composition.** All media components, including those added prior to use, are shown.

| Component | DMEM  (μM) | HPLM  (μM) | MEM  (μM) |
| --- | --- | --- | --- |
| Serine | 400 | 150 | 0 |
| Glycine | 400 | 300 | 0 |
| Cysteine | 201 | 100 | 100 |
| Glutamine | 4000 | 550 | 0 |
| Glutamate | 0 | 80 | 0 |
| Leucine | 800 | 150 | 396 |
| Threonine | 798 | 140 | 400 |
| Valine | 800 | 220 | 393 |
| Phenylalanine | 400 | 80 | 194 |
| Glucose | 25000 | 25000 | 5500 |
| Fructose | 0 | 40 | 0 |
| Folate | 9 | 2 | 2 |
| Formate | 0 | 50 | 0 |
| Uridine | 0 | 3 | 0 |
| Na-Pyruvate | 0 | 50 | 0 |
| Succinate | 0 | 20 | 0 |
| Malate | 0 | 5 | 0 |
| αKG | 0 | 5 | 0 |
| GSH | 0 | 25 | 0 |
| Supplements added to media before use | | | |
| Dialysed FBS  (A3382001, Gibco) | 9.1%  (50 ml to 500 ml) | 9.1%  (50 ml to 500 ml) | 9.1%  (50 ml to 500 ml) |
| MEM Vitamins  (11120052, Gibco) | 0 | 0 | 0.91%  (5 ml in 550 ml) |
| Glucose | 0 | 0 | Increase to 17000 |
| Glutamine | 0 | 0 | 2000 |

**SI Figures**

**Figure S1. SSP is more highly expressed in lung cancer patients with hypoxic tumours.**

**A**. Expression levels of PHGDH, PSAT1, and PSPH in TCGA LUSC patient tumour (n=486) and normal (n=50) tissue samples. **B**. Expression levels of PHGDH, PSAT1, and PSPH in TCGA LUAD patient tumour (n=483) and normal (n=59) tissue samples. **C**. Correlation of PHGDH, PSAT1, and PSPH expression with the Buffa hypoxia metagene signature in TCGA LUSC cancer cohorts (n=486). **D**. Correlation of PHGDH, PSAT1, and PSPH expression with the Buffa hypoxia metagene signature in TCGA LUAD cancer cohorts (n=483). **E**. Correlation of PHGDH, PSAT1, and PSPH expression with the Winter hypoxia metagene signature in TCGA LUSC cancer cohorts (n=486). **F**. Correlation of PHGDH, PSAT1, and PSPH expression with the Winter hypoxia metagene signature in TCGA LUAD cancer cohorts (n=483). The Gepia2 expression analysis tool was used to generate A and B. Plots in C-F were generated using the GEPIA2 correlation analysis tool. Pearson’s rho correlation coefficient (R) was calculated. **p<0.01*.

**Figure S2. PHGDH is hypoxia-inducible. A.** A549 cells were treated with <0.1% O_2_ for 4, 8, 16, 24 h, and RT-qPCR for CAIX was carried out. These are the same samples has shown in Figure 1A. **B-C.** H460 cells were treated with <0.1% O_2_ for 4, 8, 16, 24 h, and RT-qPCR for PHGDH (B) and CAIX (C) was carried out. **D**. H460 cells were exposed to <0.1% O_2_ for the times indicated, followed by western blotting for PHGDH, HIF-1α and β-actin as a loading control. **E**. Quantification of changes in PHGDH from western blots generated in part D. Band intensities were normalised to β-actin loading control.

**F, G**. The positions of hypoxia-responsive elements (HRE) (**F**) and ATF4-binding domains (**G**) were determined using the Eukaryotic Promoter Database (p-value cutoff, 0.0001). The illustrated transcription factor binding motifs are from the JASPAR CORE database ^2^. For statistical analysis, a two-tailed unpaired Student t-test was performed. **p<0.05*, ***p<0.01*.

**Figure S3. Validation and characterisation of PHGDH KO cells. A-C**. ICE (interference of CRISPR edits) analysis of CRISPR-Cas9 edited cells. Discordance between Sanger sequencing traces from an unedited parental control (orange) and the CRISPR-edited (green) cells plotted across the amplicon. The alignment and interference windows are indicated. The ICE regression score (R²) is shown. **D**. A549 parental control and PHGDH KO1 were grown in standard conditions (21% O_2_, DMEM) for 7 days, and the cell number was determined each day. The fold change increase in cell number was calculated. No significant differences in proliferation rate were measured. **E, F**. A549 parental control (**E**) and PHGDH KO1 (**F**) cells were grown in DMEM, HPLM, and MEM media under normoxia for 7 days, and the cell number was determined daily. Fold change in cell number was calculated. Proliferation curves of HPLM and MEM conditions were also used in Figure 2G and H. **G, H**. A549 parental control cells were grown in HPLM (**G**) and MEM (**H**) for 0-24 h, and PHGDH levels were determined by western blotting. The 0 h time point demonstrates PHGDH levels in A549 cells grown in DMEM. β-actin was used as a loading control. **I**. A549 parental control cells were cultured in DMEM (D), MEM (M) or MEM supplemented with increasing concentrations of serine for 24 h hours. PHGDH levels were determined by western blotting. β-actin was used as a loading control. Data represent the mean of three replicates, and error bars indicate the SEM. For statistical analysis of the proliferation data, a two-tailed paired Student *t-test* was used. For western blot data, a two-tailed unpaired Student t-test was performed. **p<0.05*, ****p<0.001*.

**Figure S4. Impact of exogenous serine and oxygen on radiosensitivity A**. A549 cells were grown in DMEM, HPLM or MEM for 20 hours and were then irradiated (0-8 Gy). 24 hours after irradiation, all media were replaced with fresh DMEM, and colonies were allowed to form. These data are the same as shown in Figures 3A, B and C. **B**. A549 (parental) and KO2 cells were grown in MEM and exposed to radiation (6 Gy) under either normoxic (N) or hypoxic (H; <0.1% O_2_) conditions, and radiosensitivity was determined by colony survival assay. IR- indicates unirradiated cells, and IR+ indicates cells exposed to 6 Gy radiation. These data are presented as survival fraction (%) relative to unirradiated controls. **C**. A549 (parental) cells were grown in DMEM and exposed to radiation (0-8 Gy) in either normoxia or hypoxia (<0.1% O_2_), and radiosensitivity was determined by colony survival assay. These data are the same as shown in Figure 3A and D. **D**. A549 (parental) cells were grown in HPLM and exposed to radiation (0-8 Gy) in either normoxia or hypoxia (<0.1% O_2_), and radiosensitivity was determined by colony survival assay. These data are the same as shown in Figure 3B and E. **E**. A549 (parental) cells were grown in MEM and exposed to radiation (0-8 Gy) in either normoxia or hypoxia (<0.1% O_2_), and radiosensitivity was determined by colony survival assay. These data are the same as shown in Figures 3C and F. Data represent the mean of three replicates, and error bars indicate the SEM. For statistical analysis, survival curves were fitted using a linear–quadratic model and compared by an extra-sum-of-squares F test. For panel B, a two-tailed unpaired Student t-test was performed. **p<0.05*, *****p<0.0001*.

**Figure S5. Loss of PHGDH in the absence of extracellular serine results in lower GSH and increased DNA damage. A.** Parental control and PHGDH KO1 A549 cells were grown in DMEM, HPLM and MEM for 16 h, under normoxia (N, 21% O_2_) or hypoxia (H, <0.1% O_2_, 16 h). Fold change in GSH levels was calculated in relation to baseline levels in cells cultured in DMEM under normoxia. A549 cells were also treated with N-ethylmaleimide (NEM) (200 μM, 6 h) in each media type to reduce GSH, as a negative control. Normoxic and NEM data presented in this panel were previously used in Figure 4A. **B-D.** A549 parental control (Con) and PHGDH KO (KO2 and KO3) cells were cultured in DMEM (**B**), HPLM (**C**), or MEM (**D**) and irradiated with 0 or 2 Gy. Representative western blots show PHGDH and γH2AX levels, with β-actin as a loading control. Data represent the mean of three replicates, and error bars indicate the SEM. For statistical analysis, a two-tailed unpaired Student t-test was performed. **p<0.05*, ***p<0.01*.

**Figure S6. PHGDH KO cells show improved survival and proliferation under hypoxia. A**. A549 parental control and PHGDH KO (KO2 and KO3) cells grown in DMEM were exposed to hypoxia (<0.1 or 2% O_2_) for 16 h. Cells were returned to normoxic conditions and colonies allowed to form. Data for Con A549 is the same as shown in Figure 5A. **B**. Loss of PHGDH was again confirmed by western blotting of the parental (Con) and PHGDH KO clones. **C**. Fold change in proliferation rates of parental control A549 cells grown in complete DMEM media with (dashed line) or without (solid line) 16 h exposure to <0.1% O_2_. **D**. Fold change in proliferation rates of PHGDH KO A549 cells grown in complete DMEM media with (dashed line) or without (solid line) 16 h exposure to <0.1% O_2_. **E, F**. A549 parental control and PHGDH KO (KO2 and KO3) cells grown in HPLM (**E**) and MEM (**F**) were exposed to hypoxia (<0.1 or 2% O_2_) for 16 h. Following the hypoxia exposure, cells were kept in HPLM or MEM for an additional 24 h and then returned to DMEM and grown under normoxia to allow colony formation. **G**. Parental control (Con) and PHGDH KO2 cells expressing an empty or overexpression vector (KO2 + PHGDH) were cultured under normoxia and hypoxia (<0.1 or 2% O_2_) for 16 h. Cells were returned to normoxic conditions and colonies allowed to form. **H**. Representative western blot showing the expression of PHGDH in parental control, PHGDH KO2 and PHGDH overexpressing cells. β-actin was used as a loading control. Data represent the mean of three replicates, and error bars show the standard error of the mean (SEM). For statistical analysis, a two-tailed unpaired Student t-test was performed. **p<0.05*, ***p<0.01*.

**Figure 7. Loss of PHGDH leads to increased HIF-1 accumulation and increased expression of HIF-target genes. A.** A549 parental control and PHGDH KO (KO1 and KO2) clones were exposed to <0.1% O_2_ for 0.5-2 h, followed by western blotting with the antibodies indicated. β-actin was used as a loading control. **B.** A549 cells and PHGDH KO2 cells were transfected with an HRE-Luciferase reporter and then exposed to hypoxia (<0.1% O_2_) for 8 hours. Hypoxic luciferase levels relative to normoxia are shown. **C, D.** A549 and PHGDH KO2 cells were exposed to hypoxia (<0.1% O_2_) for the times indicated, followed by qPCR for CAIX (**C**), GLUT1 (**D**). **E, F**. A549 and PHGDH KO1 cells were exposed to hypoxia (<0.1% O_2_) for 16 h, followed by qPCR for LDHA (**E**), VEGF (**F**). Data represent the mean of three replicates, and error bars show the standard error of the mean (SEM). For statistical analysis, a two-tailed unpaired Student t-test was performed. **p<0.05,* ***p<0.01*.
